# The diet rapidly and differentially affects the gut microbiota and host lipid mediators in a healthy population

**DOI:** 10.1186/s40168-023-01469-2

**Published:** 2023-02-11

**Authors:** Isabelle Bourdeau-Julien, Sophie Castonguay-Paradis, Gabrielle Rochefort, Julie Perron, Benoît Lamarche, Nicolas Flamand, Vincenzo Di Marzo, Alain Veilleux, Frédéric Raymond

**Affiliations:** 1 Centre Nutrition, santé et société (NUTRISS), Institut sur la nutrition et les aliments fonctionnels (INAF), École de nutrition, Université Laval, 2440, boulevard Hochelaga, Québec, G1V 0A6 Canada; 2 Canada Excellence Research Chair in the Microbiome-Endocannabinoidome Axis in Metabolic Health, Quebec, Canada; 3 Centre de recherche de l’Institut de cardiologie et de pneumologie de Québec, Faculté de médecine, Département de médecine, Université Laval, Québec, Canada; 4 Unité Mixte Internationale en Recherche Chimique et Biomoléculaire sur le Microbiome et son Impact Sur la Santé Métabolique et la Nutrition (UMI-MicroMeNu), Université Laval and Consiglio Nazionale delle Ricerche, Istituto di Chimica Biomolecolare, Via Campi Flegrei 34, 80078 Pozzuoli, (NA) Italy

**Keywords:** Endocannabinoid, Endocannabinoidome, Short-chain fatty acids, Polyunsaturated fatty acids, Metabolomics, Diet, Mediterranean, Western diet, Gut microbiota, Microbiome diversity

## Abstract

**Background:**

Bioactive lipids produced by human cells or by the gut microbiota might play an important role in health and disease. Dietary intakes are key determinants of the gut microbiota, its production of short-chain (SCFAs) and branched-chain fatty acids (BCFAs), and of the host endocannabinoidome signalling, which are all involved in metabolic diseases. This hypothesis-driven longitudinal fixed sequence nutritional study, realized in healthy participants, was designed to determine if a lead-in diet affects the host response to a short-term dietary intervention. Participants received a Mediterranean diet (MedDiet) for 3 days, a 13-day lead-in controlled diet reflecting the average Canadian dietary intake (CanDiet), and once again a MedDiet for 3 consecutive days. Fecal and blood samples were collected at the end of each dietary phase to evaluate alterations in gut microbiota composition and plasma levels of endocannabinoidome mediators, SCFAs, and BCFAs.

**Results:**

We observed an immediate and reversible modulation of plasma endocannabinoidome mediators, BCFAs, and some SCFAs in response to both diets. BCFAs were more strongly reduced by the MedDiet when the latter was preceded by the lead-in CanDiet. The gut microbiota response was also immediate, but not all changes due to the CanDiet were reversible following a short dietary MedDiet intervention. Higher initial microbiome diversity was associated with reduced microbiota modulation after short-term dietary interventions. We also observed that BCFAs and 2-monoacylglycerols had many, but distinct, correlations with gut microbiota composition. Several taxa modulated by dietary intervention were previously associated to metabolic disorders, warranting the need to control for recent diet in observational association studies.

**Conclusions:**

Our results indicate that lipid mediators involved in the communication between the gut microbiota and host metabolism exhibit a rapid response to dietary changes, which is also the case for some, but not all, microbiome taxa. The lead-in diet influenced the gut microbiome and BCFA, but not the endocannabinoidome, response to the MedDiet. A higher initial microbiome diversity favored the stability of the gut microbiota in response to dietary changes. This study highlights the importance of considering the previous diet in studies relating the gut microbiome with lipid signals involved in host metabolism.

Video Abstract

**Supplementary Information:**

The online version contains supplementary material available at 10.1186/s40168-023-01469-2.

## Introduction

Evidence points to gut microbiota dysbiosis as a hallmark of various chronic diseases [1, 2]. Indeed, several species of bacteria are associated with health conditions, as well as with dietary patterns [3–5]. Links between dietary intakes and metabolic disorders have been observed, as well as between these disorders and microbiota components [6, 7]. However, the causality of these interactions is still nebulous as microbiota changes are closely linked with diet and could be either a cause or a consequence of metabolic disorders [8].

Bioactive lipids produced by human cells or by the gut microbiota might play an important role in metabolic alterations [9, 10]. Gut bacteria produce short-chain fatty acids (SCFAs) and branched-chain fatty acids (BCFAs) from dietary fibers through carbohydrate and protein fermentation, respectively [10]. These metabolites serve as an energy source for colonocytes and have cellular signalling functions, including the modulation of inflammation and motility of the intestine, lipid, and glucose metabolism, as well as satiety [11, 12]. In the host, the endocannabinoidome (eCBome) is a relatively new player in the response of the individual to the diet and in the regulation of both gut microbiome function and metabolic control [13, 14]. The eCBome is a complex lipid signalling system that comprises receptors and mediators involved in most of those metabolic functions that are altered in metabolic diseases [15]. The mediators are ultimately derived from fatty acids, which, especially for the polyunsaturated fatty acids (PUFAs), are only found in dietary sources such as fish, vegetable oils, seed, and nuts [16]. They belong to various families of long-chain fatty acid amides and esters, such as the *N*-acyl-ethanolamines (NAEs) and 2-mono-acyl-glycerols (2-MAGs), which in turn include the two endocannabinoids, anandamide (*N*-arachidonoyl-ethanolamine, AEA) and 2-arachidonoyl-gycerol (2-AG), respectively [17]. Recent articles have shown the importance of dietary fat intake as a determinant of circulating NAE and 2-MAG levels and highlighted the interaction between the eCBome and the gut microbiota [18–21].

Long-term food intake is known to deeply influence the gut microbiome [22]. Microbiome composition, in association with specific diet components, has been associated to cardiometabolic risk [23] and recent work suggests that the effectiveness of cardiometabolic risk protection by the Mediterranean diet (MedDiet) is influenced by microbiome composition [4]. In addition, long-term dietary patterns similar to a MedDiet have been associated to specific properties of microbiome composition, including modulation of keystone species, and to lower intestinal inflammation [24, 25]. Still, meta-analysis of the literature on the effects of the MedDiet on the gut microbiome did not show consensus between studies [26]. Although long-term diet is a driver of gut microbiota composition, studies also show that the latter also responds to short-term dietary changes [3]. In a mouse model, 3 days of high-fat, high-sucrose diet was sufficient to modulate bacterial genera in the jejunum, ileum, and caecum, with intensifying responses as the treatment duration increased [20]. In a cohort of healthy female athletes, 7 days of MedDiet or CanDiet had a modest effect on microbiome composition, but strongly modulated the impact of aerobic exercise on the immediate response of plasma eCBome mediators and SCFAs [27], suggesting a divergent scale in the response to the diet of different biological systems. Resilience of the gut microbiome, sometimes associated with microbial diversity [28], might also play a role in the response to dietary interventions [29].

**Fig. 1:**
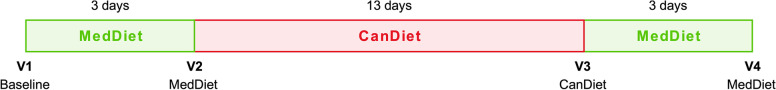
Schematic representation of study design

In this work, we aimed to experimentally determine the impact of the previous diet on the eCBome and microbiome response to a short-term MedDiet intervention. Our study was designed to investigate the effect of a lead-in diet to the response of participants to a 48-h MedDiet intervention. Thus, we conducted a longitudinal controlled feeding study in a cohort of healthy volunteers to gain experimental insight on the interaction between diet, plasma lipid mediators, and the gut microbiome. Blood and fecal samples were collected: (1) at baseline, (2) after 48 h of MedDiet, which is rich in polyunsaturated fatty acids, fibers and polyphenols, (3) after 13 days of average Canadian diet (CanDiet) and, finally, (4) after a second 48 h of the same MedDiet intervention (Fig. 1). Our primary objective was to determine if the lead-in CanDiet affected the response to MedDiet of plasmatic lipid mediators, namely the eCBome, SCFAs, and BCFAs, and of fecal microbiota composition. We compared the scale of the response of these variables to the change in diet and investigated the potential interactions between lipid mediators and the microbiome in response to diet. We specifically looked for the consequences of the lead-in diet on changes in the response to MedDiet. The influence of the initial microbiome diversity of the participants on bioactive lipids and microbiome modulation was also assessed. This experimental, hypothesis-driven clinical trial provides in vivo insights into the interactions between the diet, plasma lipid mediators, and the human gut microbiome, with potential implications for metabolic disorders and their comorbidities.

## Material and methods

### Controlled feeding intervention

This fixed sequenced study is summarized in Fig. 1. The first 3 days, a Mediterranean diet (MedDiet) was provided, then a Canadian diet (CanDiet) for 13 days and finally a Mediterranean diet (MedDiet) for 3 days. Details about the composition of the diets are given in Table 1. The CanDiet was designed to reflect the current Canadian macronutrient intake while avoiding short-term nutritional deficiencies. The MedDiet was characterized by greater intake of fruits and vegetables, plant-based proteins and grains. It contained higher amounts of monounsaturated fatty acids (MUFAs), omega-3 PUFA, and fibers, with less saturated fatty acids (SFAs) and red meat [30]. Subjects were instructed to consume only the foods and beverages provided to them, which corresponded to their estimated energy needs. Energy needs for each subject were estimated by averaging the energy requirements estimated by a validated web-based 24-h dietary recall (R24W) completed on three occasions, and energy expenditure obtained with Harris–Benedict formula [31–33]. Healthy eating index (HEI) was calculated based on these R24W [34]. A checklist was provided to the participants to identify the food that had not been consumed. A form was provided to indicate food items that had been consumed in addition to the food provided. One participant was removed from the study due to non-compliance.

**Table 1:** Composition of the MedDiet and CanDiet diets in the study

	MedDiet	CanDiet
HEI score	80	65
Energy (kcal)	2502	2500
Energy from carbohydrates (%)	46.81	48.42
Total dietary fibers (g)	45.48	19.08
Energy from proteins (%)	15.75	17.46
Animal proteins (g)	56.02	73.00
Plant proteins (g)	44.27	33.44
Energy from lipids (%)	35.11	34.05
Energy from saturated fatty acids (%)	6.20	15.19
Energy from monounsaturated fatty acids (%)	20.35	11.14
Energy from polyunsaturated fatty acids (%)	6.24	4.90
Linoleic acid (18:2) (g)	15.62	11.49
Alpha-linolenic acid (18:3) (g)	1.60	1.08
Arachidonic acid (20:4) (g)	0.13	0.14
Eicosapentaenoic acid (20:5) (g)	0.20	0.01
Docosapentaenoic acid (22:5) (g)	0.06	0.01
Docosahexaenoic acid (22:6) (g)	0.45	0.04

### Ethics

Written informed consent of participants was obtained. The study was approved by the Laval University Ethics Committee (2017-328 and 2018-262) and registered in the ClinicalTrials.gov registry (NCT03783260). The longitudinal controlled feeding study was conducted between December 2018 and March 2019 at the Institute of Nutrition and Functional Foods (INAF) in Quebec City, Canada.

### Sample characteristics

Twenty-one healthy young adults from 20 to 34 years old composed of 11 women and 10 men with general good health and a body mass index between 18.5 and 30 kg/m^2^ completed the study successfully (Table 2). Subjects with enteropathies, alcohol consumption exceeding the Canadian recommendation for men (> 15 drinks/week) and women (> 10 drinks/week), active tobacco usage, consumption of dietary supplements (e.g., multivitamins, omega-3, probiotics), weight change (±5 kg) in the last 6 months, having taken antibiotics in the last 3 months, and pregnant and/or breastfeeding women were not eligible.

**Table 2:** Anthropometric and metabolic characteristics of study participants (*n*=21)

	Women (*n*=11)		Men (*n*=10)	
	Mean	Range	Mean	Range
Age	26.82	20–34	23.20	20–29
BMI (kg/m^2^)	21.60	20.1–24.1	22.22	20.4–25
WC (cm)	71.50	65.8–78.6	78.61	74–87.2
Fasting glucose (mmol/L)	4.47	4–4.9	4.49	4–4.9
Triglycerides (mmol/L)	0.82	0.61–1.78	0.86	0.62–1.11
HOMA-IR	1.09	0.38–2	1.24	0.4–2.27
HEI score	64.94	39.5–87	56.36	37.5–74.4

### Sample collection

Overnight fasting blood samples as well as fecal samples were collected on the morning of each dietary change (Fig. 1). Fecal samples were immediately aliquoted and frozen by the participant. Samples at V2 and V4 were taken after 48 hours of MedDiet. A posteriori data analysis confirmed that all fecal samples were collected between waking up and noon.

### Circulating lipid quantification

Levels of PUFAs, NAEs, and MAGs in plasma samples (200 μL) were measured using high-performance liquid chromatography coupled to tandem mass spectrometry (LC–MS/MS) as previously described [35]. It allowed the quantification of NAEs including *N*-arachidonoylethanolamine or anandamide (AEA), *N*-palmitoyl-ethanolamine (PEA), *N*-oleoyl-ethanolamine (OEA), *N*-linoleoyl-ethanolamine (LEA), *N*-eicosapentaenoyl-ethanolamine (EPEA), and *N*-docosahexaenoyl-ethanolamine (DHEA), as well as MAGs including 1/2-Arachidonoylglycerol (AG), 1/2-palmitoyl-glycerol (PG), 1/2-oleoyl-glycerol (OG), 1/2-linoleoyl-glycerol (LG), 1/2-eicosapenaenoyl-glycerol (EPG), 1/2-docosapentaenoyl-glycerol (DPG), and 1/2-docosahexaenoyl-glycerol (DHG). In the case of polyunsaturated 1- and 2-MAGs, the data are presented as 2-MAGs but they represent the combined signals from the 1(3)- and 2-isomers because the 1(3) isomers are most likely generated via acyl migration from the 2-isomers. PUFAs, including arachidonic acid (AA), docosahexaenoic acid (DHA), docosapentaenoic acid (DPA), and eicosapentaenoic acid (EPA), were also measured. Values of 0 were replaced by the value of half the detection limit for each metabolite. Quantification of SCFAs and BCFAs was performed on a GC-FID system at INAF analytical platform as previously described [27].

### 16S rRNA gene sequencing

Stool bacterial DNA was extracted using the QIAamp DNA Stool Kit (QIA- GEN, CA, USA), and amplification of the V3–V4 region was performed using the primers 341F (5′-CCTACG GGNGGCWGCAG-3′) and 805R (5′-GACTACHVGGGTATCTAATCC-3′) (Illumina, CA, USA) as previously described [20]. Briefly, libraries were purified using magnetic beads (Axygen Biosciences, CA, USA) and quality assessed (Agilent Technologies, CA, USA). High-throughput sequencing (2 × 300 bp paired end) was performed on Illumina MiSeq. Sequences were processed using the Dada2 package (Version 1.10.1), and associations to bacterial taxa were obtained using the Silva v132 reference database [36, 37]. Data have not been rarefied. The sequence counts per sample median are 46,904, mean 46,442, minimum 21,134, and maximum 79,290 (Figure S1). Sequence variant tables were transformed at each taxonomic level in relative abundances. Statistical analyses such as the mixed linear-effect models (LME) and the multiple factor analysis (MFA) were only performed on taxa that were at more than 1% in at least one sample. Raw sequencing reads are available in SRA (PRJNA810015).

### Statistical approach

All figures and statistical analyses were performed using R studio software (RStudio 1.2.1335, R version 4.1.3). Mixed linear-effect models (LME) including random individual effect was used to identify lipid mediators and microbiome taxa at more than 1% in at least one sample that were influenced by diet. The genus taxonomic level was selected for analysis, as it best explained the variability between our samples based on a multiple factor analysis (MFA) made on all taxonomic levels (Figure S3). The primary and secondary objectives were to determine if lead-in diet (regular diet different between participants or standardized CanDiet) affected plasma lipid mediators (primary) and microbiome (secondary) response to MedDiet intervention. Plasma lipid concentrations have been normalized using ranked values fitted into LME and significance has been tested by analysis of variance (ANOVA) with random effects nested within participants. False discovery rate-corrected *p*-value lower than 0.05 was considered statistically significant. False discovery rate (FDR) correction on *p*-values was made with the function p.adjust of the stats package. Principal component analysis (PCA) and multiple factor analysis (MFA) were made with the FactoMineR package [38]. PCA and MFA plots were made with the factoextra package. MFA microbiome analysis included all taxonomic ranks to improve interpretability, and taxa representing 1% in at least one sample were included in the analysis. Permutational multivariate analysis of variance (PERMANOVA) has been made using Adonis function of the package vegan with 100,000 permutations. Hierarchical clustering from the MFA or PCA analysis were made using the HCPC function of the hierarchical clustering on FactoMineR package. Variables significantly different between clusters were also obtained by the HCPC function. The function binom.test of the stats package was used to test the distribution within clusters of samples from MedDiets or CanDiet and baseline. Simpson’s and Shannon diversity indices were calculated on all sequence variants using the vegan package [39]. Most plots were drawn with ggplot2 package and statistical analyses for geom_boxplot were calculated by the function stat_compare_means from the ggpubr package. Barplots were drawn with barplot2 function from the gplots package. Colored bars below dendrograms were drawn using the dendextent rpackage [40]. Heatmaps were drawn using pheatmap package, and correlation plots were drawn with corrplot package. Spearman correlations were calculated using the function cor.test from the stats package. The stats package function lm was used for the regression lines and dist to calculate Euclidean distance. The interaction network was made with Cytoscape Version 3.8.0.

## Results

### Short-term dietary intervention determines plasma lipid mediators

**Fig. 2:**
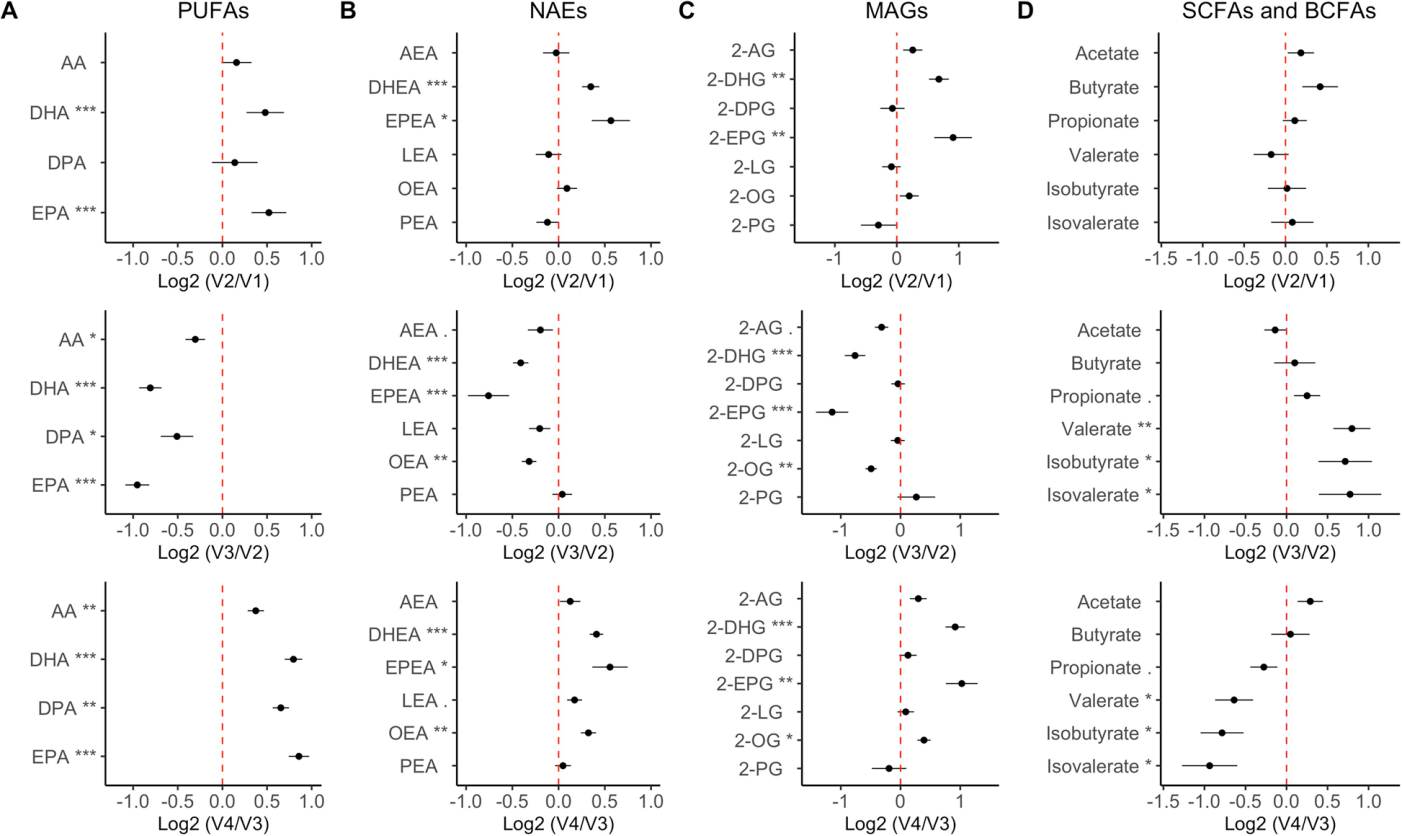
Response of plasma bioactive lipids to the dietary intervention. Panels show results for **A** polyunsaturated fatty acids (PUFAs), endocannabinoidome mediators classes, **B** N-acylethanolamines (NAEs), and **C** monoacylglycerols (MAGs) and **D** short-chain fatty acids (SCFAs) and branched-chain fatty acids (BCFAs). The graph represents the mean with the standard error of the log2 ratio between the plasmatic lipid concentrations at MedDiet (V2) vs Baseline (V1) (top panel), CanDiet (V3) vs MedDiet (V2) (middle panel), and MedDiet (V4) vs CanDiet (V3) (bottom panel). The red line drawn at 0 represents no change in the metabolite. Lipid concentrations have been normalized using ranked values fitted into mixed linear-effect model (LME) and differences between visits have been tested by analysis of variance (ANOVA). *P*-values have been corrected by false discovery rate (FDR). Significance was set at *p*<0.1 (.), *p*<0.05 (*), *p*<0.01 (**), and *p*<0.001 (***). Names of the molecules are arachidonic acid (AA), docosahexaenoic acid (DHA), docosapentaenoic acid (DPA), eicosapentaenoic acid (EPA), anandamide (AEA), *N*-docosahexaenoyl-ethanolamine (DHEA), *N*­eicosapentaenoyl-ethanolamine (EPEA), *N*-linoleoyl-ethanolamine (LEA), *N*-oleoyl-ethanolamine (OEA), *N*-palmitoyl-ethanolamine (PEA), 2-arachidonoyl-glycerol (2-AG), 2-docosahexaenoyl-glycerol (2-DHG), docosaepentaenoic-glycerol (2-DPG), 2-eicosapentaenoyl-glycerol (2-EPG), 2-linoleoyl-glycerol (2-LG), 2-oleoyl-glycerol (2-OG) and 2-palmitoyl-glycerol (2-PG)

We assessed the effect of short-term MedDiet dietary interventions with and without a lead-in diet stabilization period with a CanDiet (Fig. 2). As expected, the plasma concentrations of polyunsaturated fatty acids increased in response to both MedDiet interventions compared to baseline and CanDiet (Fig. 2A). Increases after both MedDiet (V2 and V4) were also observed for several eCBome mediators including the NAEs, DHEA, and EPEA (Fig. 2B), and the 2-MAGs, 2-DHG, and 2-EPG (Fig. 2C). BCFAs and SCFAs were not significantly altered by the first MedDiet intervention (Fig. 2D). However, propionate, valerate, isobutyrate, and isovalerate were significantly increased after the CanDiet and then decreased upon the second MedDiet. There was no significant difference between the levels of bioactive lipids between MedDiet at V2 and V4, but analysis of the latter indicates that diet stabilization improved reproducibility of the metabolite response. Overall, the fold variation of metabolites between the second MedDiet and the CanDiet (V4/V3), and between the CanDiet to the first MedDiet (V3/V2) were the exact opposite, indicating a direct influence of diet on these metabolites. Overall, the lead-in diet did not affect the response of bioactive lipids to the MedDiet as these metabolites responded to short-term changes in diet, although with BCFAs the response was stronger and less variable after the second MedDiet intervention.

### Gut microbiota responds to dietary changes within 48 h

**Fig. 3:**
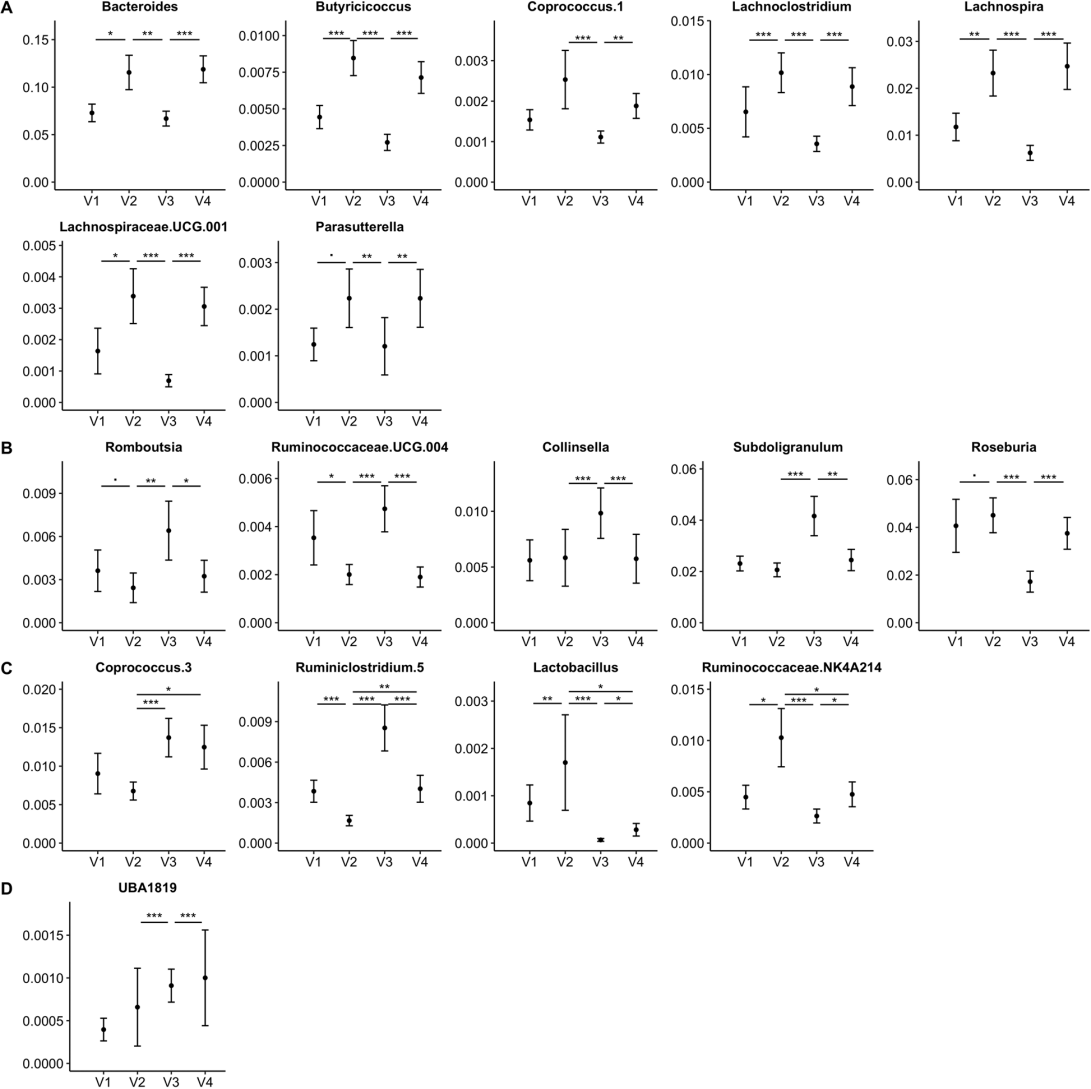
Response of gut microbiome to the dietary intervention. Mean relative abundance with standard error of the microbiota genera significantly affected by the dietary interventions are displayed. **A** Microbiota genera increased by the MedDiet, **B** are modulated by CanDiet in a manner that was reversible by the MedDiet, **C** are modulated by the CanDiet and do not recover with the MedDiet, or **D** others. Relative abundance of the microbiota genera that were at more than 1% in at least one sample has been normalized using ranked values fitted into mixed linear-effect model (LME) and differences between visits have been tested by analysis of variance (ANOVA). *P*-values have been corrected by false discovery rate (FDR). Significance was set at *p*<0.1 (.), *p*<0.05 (*), *p*<0.01 (**), and *p*<0.001 (***)

We also investigated the fecal microbiome response to short-term MedDiet dietary interventions with and without a lead-in diet stabilization period with CanDiet (Fig. 3, Supplementary Table S1). Simpson’s and Shannon microbiome diversity were significantly higher after dietary intervention compared to baseline diversity (Figure S2A-B). All significantly modulated taxa had baseline relative abundances lower than 5%, except for *Bacteroides*. Three patterns of microbiota response were identified. First, MedDiet interventions led to a reproducible increase of seven genera independently of lead-in diet: *Bacteroides*, *Butyricoccus*, *Coprococcus.1*, *Lachnoclostridium*, *Lachnospiraceae UCG 001*, *Parasutterella*, and *Lachnospira* (Fig. 3A). Second, CanDiet modulated genera in a manner that was reversible by the MedDiet, including *Romboutsia*, *Ruminococcaceae UCG 004*, *Roseburia*, *Subdoligranulum*, and *Collinsella* (Fig. 3B). Third, and most important, taxa modulated by the CanDiet did not return to their initial relative abundance after the second MedDiet (Fig. 3C). For instance, the relative abundance of *Coprococcus 3* and *Ruminiclostridium 5* was significantly different at the end of each MedDiet intervention period (V2, V4). Thus, the increase induced by the CanDiet stabilization was still observed at V4 for these taxa. Similarly, *Ruminococcaceae NK4A214* and *Lactobacillus* were significantly decreased by the CanDiet (V2, V3) but did not recover after the second MedDiet (V4) (Fig. 3C). Finally, unclassified *Faecalibacterium* UBA1819 increased throughout the study (Fig. 3D). These results indicate that gut microbiota responded to the MedDiet within 48 h and that, for some taxa, a 2-week CanDiet affected the gut microbiota response to the MedDiet.

### Interindividual differences in microbiota response to dietary interventions

**Fig. 4:**
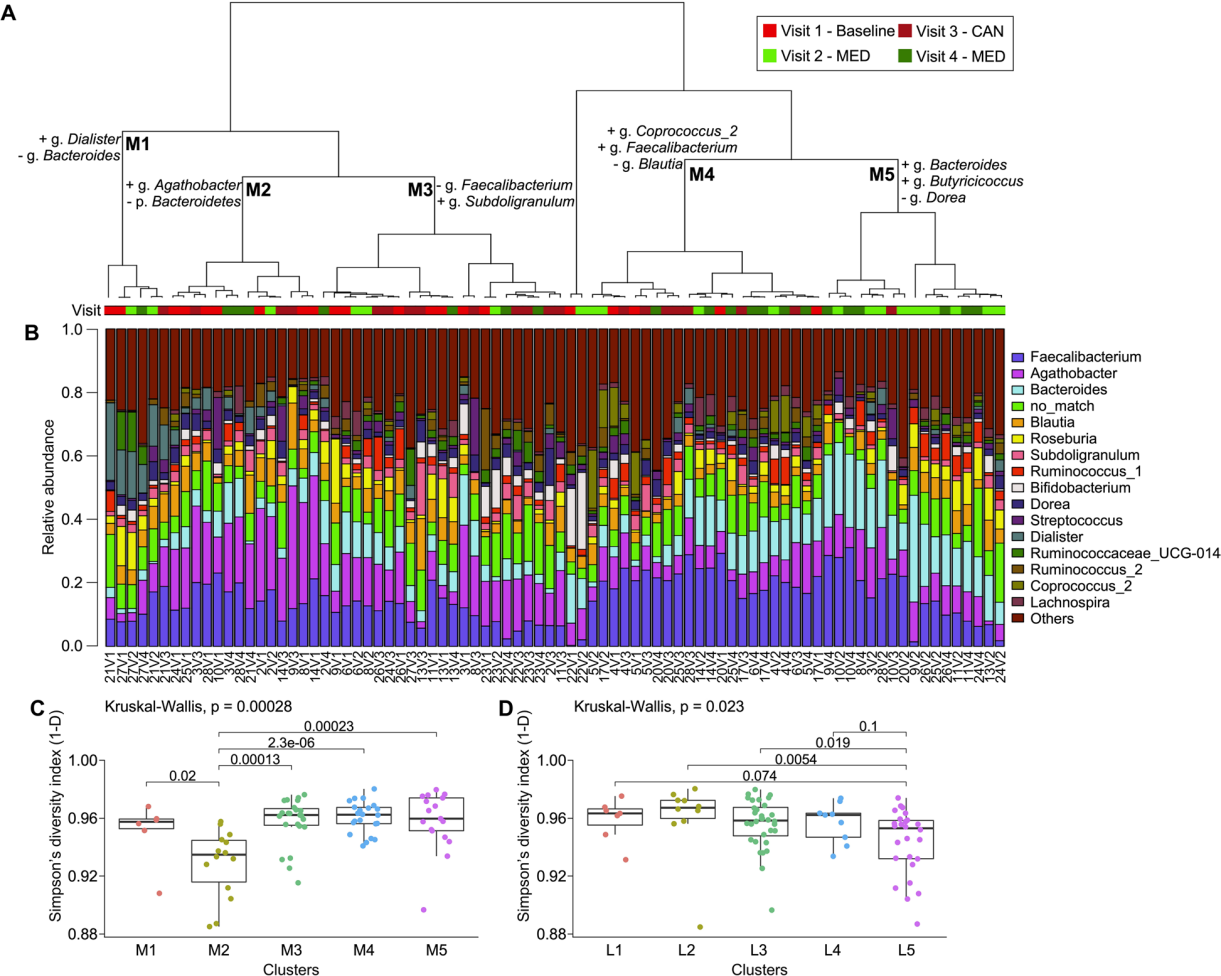
Clustering of microbiota taxonomical profiles. **A** Hierarchical clustering on multiple factor analysis of the microbiota profile of individuals at different visits. Variables contributing to the difference between the clusters are displayed on the dendrogram. Horizontal colored bar below the dendrogram represents the visit associated with sample. **B** Barplot showing the relative abundance of the 16 more abundant microbiota genera. Microbiota alpha diversity measured on all sequence variants by Simpson’s index (1-D) between **C** microbiota clusters and **D** bioactive lipid clusters

It is known that the gut microbiota of every individual harbor unique features and that they can respond differently to dietary interventions [41, 42]. Since the baseline microbiota of participants might reflect their long-term diet or other underlying conditions, we investigated interindividual differences in microbiome composition to determine if subsets of participants could have specific response to dietary interventions. To do so, we compared microbiome profiles using multiple factor analysis (Figure S3), with taxonomical ranks included as different groups of variables, followed by hierarchical clustering of the resulting variance decomposition [28] (Fig. 4). Samples were grouped by dietary interventions (chi-squared, *p*=0.003) (Figure S3D, Fig. 4A). Indeed, from the 5 main clusters identified, two were enriched in a specific diet. Cluster M2 was significantly associated with baseline samples (7/14, *p*=0.04) and showed significantly lower microbiome diversity than the four other clusters (Fig. 4C). Cluster M5 was enriched in MedDiet samples (16/17, *p*=0.0003). By contrast, cluster M1 and cluster M4 contained three to four visits of the same participant, indicating limited microbiome modulation by the diet for these individuals (Figure S4). The microbiota of these participants seemed to be less affected by the diet and more by interindividual characteristics compared to the other clusters. A similar methodology applied to bioactive lipids showed clustering mostly associated with diet (Figs. S5 and 4D). These results indicate that individual microbiomes might respond differently to short-term dietary intervention, although bioactive lipids are mostly associated with recent diet composition.

### Microbiome response to diet, but not the host bioactive lipid response, is associated to initial microbiome diversity

**Fig. 5:**
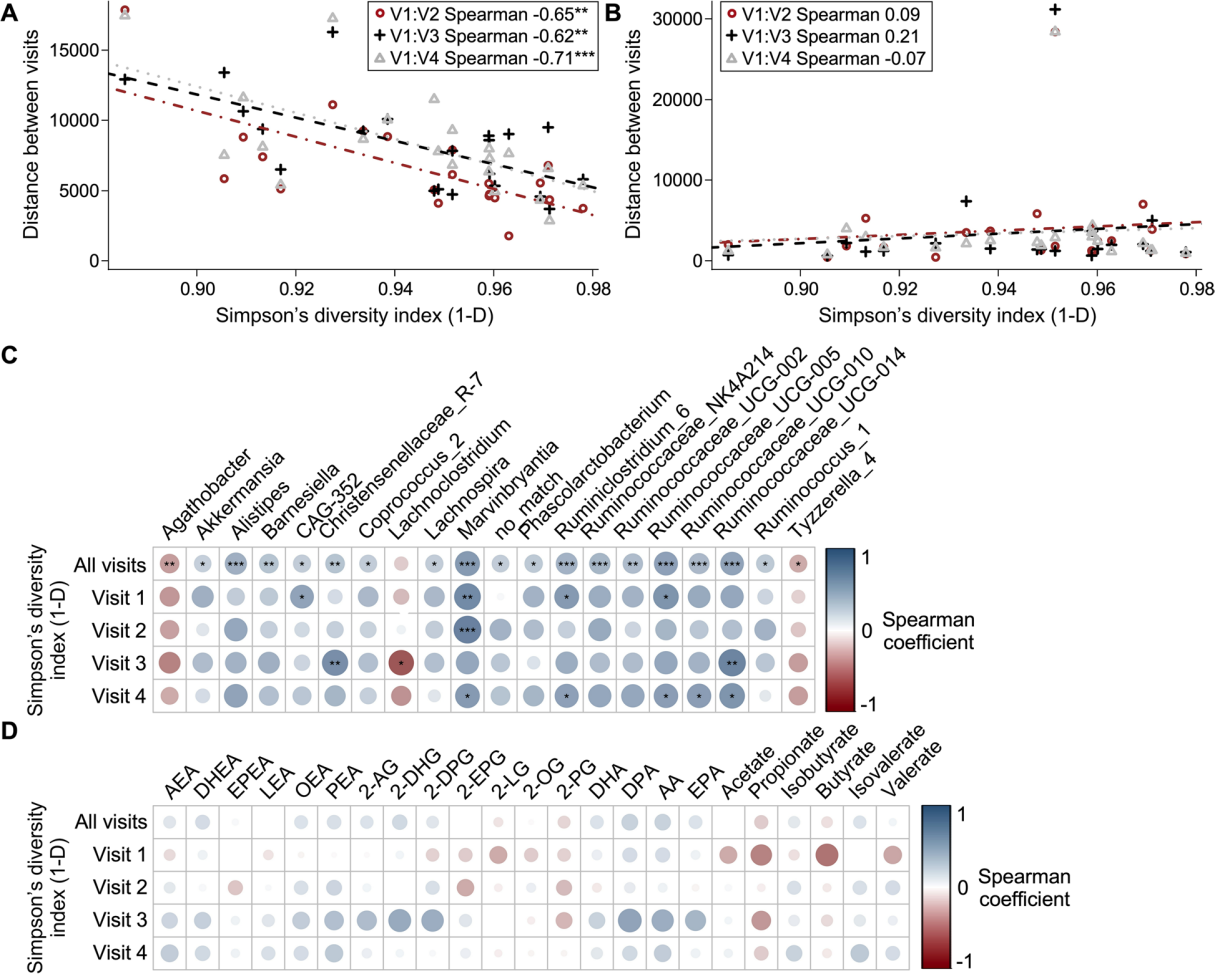
Microbiota modulation is associated with initial microbiome diversity. Euclidean distance of **A** microbiota profiles including all sequence variants or **B** lipidic profile between baseline and each visit of the study for each participant in relation with their alpha diversity at baseline. Regression lines were drawn for each visit. Slope of line (lm) and Spearman correlations were considered significant only for microbiota distance measurements with *p*-values < 0.01. Spearman coefficients are also significant and are displayed on the legend. Red represents the distance between the microbiota profile of baseline (V1) and the first MedDiet (V2), black of baseline (V1) and CanDiet (V3), and gray of baseline (V1) and the second MedDiet (V4). **C** Heatmap illustrating the FDR-corrected Spearman correlations between the gut microbiota genera that were at more than 1% in at least one sample and gut microbiota diversity calculated on all sequence variants represented by Simpson’s index (1-D) at each visit and combining the four visits. **D** Heatmap illustrating the FDR-corrected Spearman correlations between plasmatic lipid concentrations and gut microbiota diversity calculated on all sequence variants represented by Simpson’s index (1-D) at each visit and combining the four visits. Significance was set at *p*<0.05 (*), *p*<0.01 (**), and *p*<0.001 (***)

Gut microbiota diversity is believed to be associated with microbiota stability and resilience [43]. Thus, we investigated if the Euclidean distances between the microbiome profiles at each visit for each participant were correlated with their baseline microbiome indices (Simpson’s index). Overall, microbiome modulation was inversely correlated to the baseline microbiome diversity of the participants (Figs. 5A and S2C-D). Hence, individuals with greater initial microbiota diversity had smaller distances between visits. This finding supports the hypothesis that microbiota diversity is associated with microbiota stability and that this hypothesis is valid for short-term changes in the diet. A similar analysis was performed on plasma lipids, but we observed no significant correlations between microbiome diversity and the metabolite profile distances from initial metabolome (Fig. 5B). As a control, we investigated if baseline microbiome diversity was correlated with baseline healthy eating index (HEI), which was not the case (*p*=0.26, Spearman). In addition, no taxa or metabolite was significantly correlated with HEI after FDR correction (Figure S6).

### Dynamic associations between diet, microbiome diversity, and bioactive lipids

The stability of the interaction between microbiome diversity, specific taxa, and plasma metabolites with different diets was investigated by evaluating the correlations of microbial diversity with other variables at the four visits and using all data. Genera from the gut microbiota were consistently correlated with microbial diversity across the different visits for numerous taxa, highlighting the stability of the association between microbiota composition and diversity, even when changing diet (Fig. 5C). A notable exception to this general rule was *Lachnoclostridium*, which was negatively correlated with diversity only after CanDiet. Of the genera significantly associated with baseline microbiome diversity, only *Lachnoclostridium* and *Lachnospira* were significantly modulated by the diet (Fig. 3). No significant correlations between the microbiota diversity and plasma metabolites were seen after FDR correction (Fig. 5D). Interestingly, although not statistically significant, correlations between metabolites and microbiota diversity were stronger with both baseline (V1) and CanDiet (V3) compared to MedDiets (V2, V4). The difference between gut microbiota and plasma metabolite correlations with microbiome diversity highlight a dynamic association of plasma eCBome mediators, SCFAs, and BCFAs with the diet, in opposition to the stability of the association of gut bacteria with microbiome diversity at the time frames of this study.

### 2-MAGs and BCFAs have distinct correlations with the microbiome

**Fig. 6:**
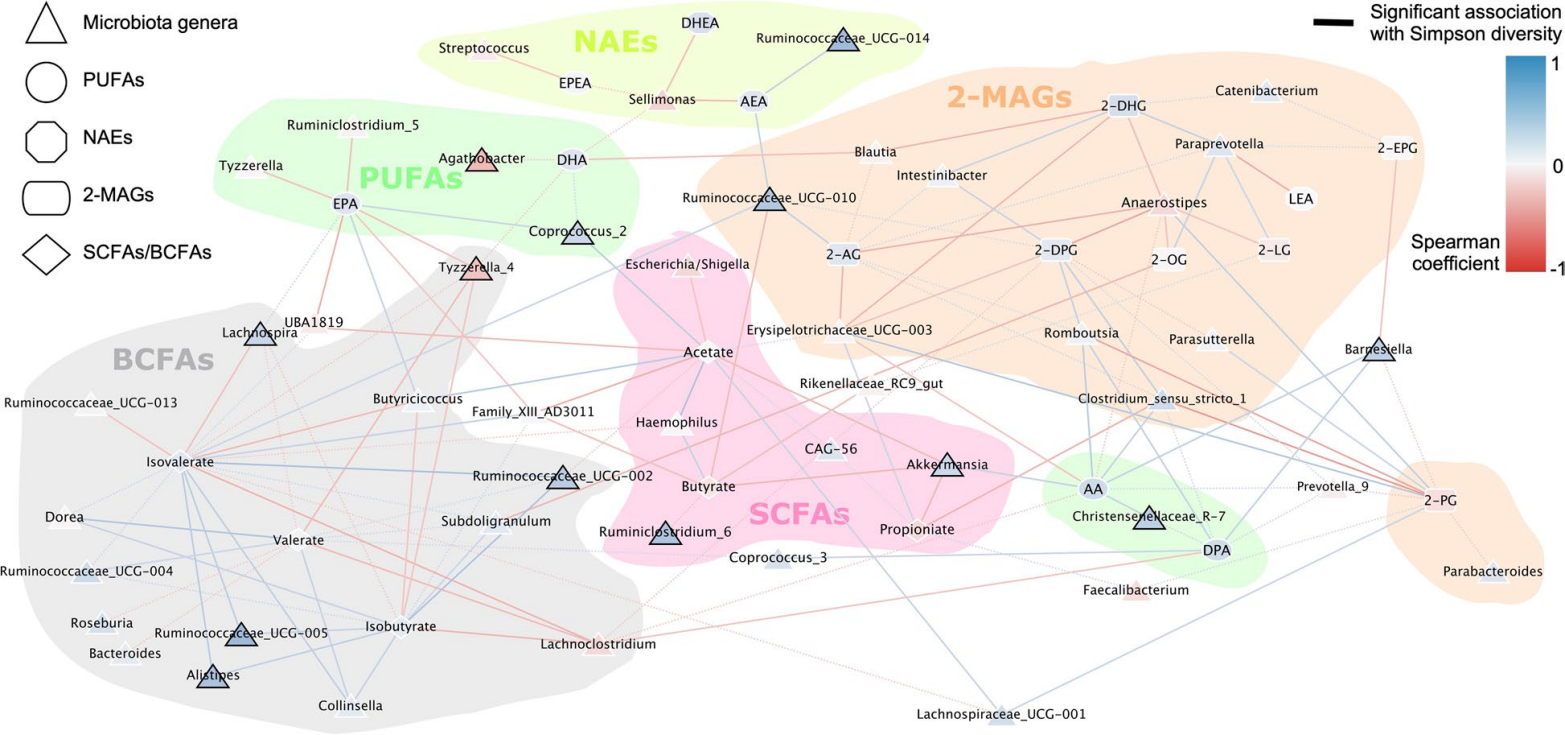
Correlation network between microbiota and bioactive lipids show distinct clusters associated with BCFAs and 2-MAGs. Network representing associations between gut microbiota genera that were at more than 1% in at least one sample and lipids based on Spearman correlations. Dotted lines represent significant correlations with a *p*-value < 0.01 and straight lines represent significant correlations after FDR with a *p*-value < 0.05. Color of the edge indicates the strength of positive (blue) or negative (red) correlation. Nodes are colored based on their association with microbiota diversity measured on all sequence variants by Simpson’s index (1-D) and nodes with significant correlations after FDR (*p*<0.05) have black border. Colored circles were drawn by hand to highlight subnetworks dividing molecules by lipid classes. Taxa were included in the group with which they had the most significant correlations. In case of a tie between two or more groups, genera were not circled

To further investigate the relation between gut bacteria and bioactive lipids, we generated a correlation network between microbiota genera relative abundances and plasma metabolite concentrations (Fig. 6). Our first general observation from this network was that lipid mediators belonging to the same chemical families were grouped together within the network, with a large subnetwork associated with 2-MAGs and another with BCFAs.

In the 2-MAGs subnetwork, *Paraprevotella* and *Anaerostipes* played central roles by correlating positively and negatively, respectively, with 2-AG, 2-DHG, 2-OG, 2-LG, and 2-EPG. These 2-MAGs were connected by positive correlation with taxa such as *Intestinibacter*, *Catenibacterium*, *Romboutsia*, and *Clostridium sensu stricto 1. Erysipelotrichaceae* was negatively associated with 2-AG, 2-DPG, and 2-DHG. Interestingly, 2-PG was separated from the subnetwork, having opposite association with gut microbiota genera compared to the other 2-MAGs. NAEs did not have high connectivity within the correlation network.

High shared connectivity was observed between bacteria and isobutyrate, isovalerate, and valerate, the most significant correlations of which were also negatively correlated with microbiome diversity. Plasma levels of valerate, isobutyrate, and isovalerate were significantly modulated by the diet and were correlated with genera also modulated by the diet (*Lachnoclostridium*, *Coprococcus*, *Bacteroides*, and *Butyricicoccus).* Taxa positively correlated with butyrate or acetate were in general negatively correlated with BCFAs. Plasmatic concentrations of the short-chain fatty acids acetate, butyrate, and propionate were negatively correlated with the genus *Akkermansia.* Plasma levels of acetate and butyrate were not modulated by the diet and showed significant correlation with taxa that were also not correlated with the diet, indicating that the production of these metabolites and associated microbiota were generally not affected by the short-term dietary intervention with the MedDiet.

Overall, taxa correlated with eCBome mediators were not significantly modulated by diet or consistently correlated with microbiome diversity, suggesting that their association with eCBome modulation could be independent of these two factors. Taxa correlated with SCFAs and BCFAs were significantly modulated between the diets (Fig. 2), and some of these taxa were correlated with microbiome diversity, suggesting that diet may influence this association. These observations are consistent with the nature of eCB and congeners, which are generally produced by the host, and of SCFAs/BCFAs, which are generally produced by the microbiome.

## Discussion

The present study aimed to define how a lead-in diet affects the response of gut microbiome and lipid mediators to a short-term MedDiet using a controlled feeding protocol in healthy subjects. It allowed to evaluate how the gut microbiota, the eCBome, and short-chain and branched-chain fatty acids adapted to dietary changes in a dynamic way. We compared the short-term effect of a MedDiet in a sample of individuals with different baseline dietary habits with that obtained, in the same individuals, following instead a 13-day stabilization period with a CanDiet. Our results showed no significant difference in the response of plasma bioactive lipids between the two MedDiet interventions. In fact, for PUFAs, MAGs, NAEs, SCFAs, and BCFAs, the fold change induced by the CanDiet compared to the first MedDiet was almost the exact opposite of the fold change induced by the second MedDiet compared to the CanDiet. However, the effect on BCFAs appeared to be stronger and affected by less variability after the MedDiet intervention that followed the CanDiet. The eCBome lipid profile appeared to be mostly determined by dietary intake, rather than the lead-in diet, and this effect was immediate and consistent. It must however be emphasized that this was observed in a young and healthy cohort submitted to short-term dietary interventions, and the picture could be different in individuals with metabolic disorders or other conditions in which these lipids have been implicated. In comparison with bioactive lipids, the response of the microbiome followed taxa-specific patterns, with some bacteria responding directly to short-term diet while others were influenced by the lead-in diet. Therefore, the main finding of this manuscript is that, in healthy individuals, the lead-in diet significantly and qualitatively affects the response to a 48-h MedDiet intervention of specific microbiome bacteria but not that of some bioactive lipids produced by the host.

The initial microbiome alpha diversity was highly correlated with gut microbiome modulation between diets, but not with the distance in plasma metabolites between the visits. We observe that a microbiota with a higher baseline alpha diversity was more resistant to dietary interventions, and hence more stable over time. A similar impact of diversity on the response to antibiotics has also been reported [28], as well as the association of microbiome instability with metabolic diseases and other conditions [44–47]. A stable microbiota would be more resistant to disturbances, and thus to dysbiosis. Therefore, microbiota stability would be one of the factors explaining the benefits of high gut microbiota diversity.

Lipid mediators measured in plasma were similarly modulated after the two MedDiets, indicating a direct link between diet and plasma metabolites. Monoacylglycerols 2-DHG, 2-EPG, and 2-OG were increased in MedDiet compared to CanDiet. These three mediators, along with 2-AG and 2-LG, were positively correlated with *Paraprevotella*. This bacterial genus is found in low abundance in T2D and preDM patients compared to healthy individuals [48]. In a study looking at metabolic status of obese children and adolescents, *Paraprevotella* has been positively associated with serum IL-6 [49], a proinflammatory factor with functions maintaining intestinal homeostasis [50]. It is also known that cannabinoid receptor 2 (CB_2_) activation by AEA or 2-AG may induce IL-6 secretion [15, 51]. Indeed, trends of positive correlation between IL-6 and 2-AG have been observed [52]. The positive association we show between *Paraprevotella* and 2-MAGs is coherent with these previous observations, while also supporting the hypothesis of an interaction between the intestinal microbiota and the endocannabinoid system. In addition, 2-MAGs (2-AG, 2-DPG and 2-DHG) and PUFAs (AA and DPA) positively correlated with *Intestinibacter*, *Romboutsia*, or *Clostridium sensu stricto 1*, three bacterial genera negatively associated with T2D prevalence [53]. These correlations are consistent with the roles of unsaturated long-chain 2-MAGs including 2-AG, 2-DPG, and 2-DHG, as they may increase insulin sensitivity and decrease glucose intolerance through activation of CB_2_, TRPV1, and other targets [20, 54, 55].

*N*-acylethanolamines DHEA, EPEA, and OEA also showed an increase in MedDiet compared to CanDiet. Observational cohort has shown that levels of 2-DHG, 2-EPG, DHEA, and EPEA correlate with dietary intake of DHA or EPA [21], a relation we also observe throughout the current intervention, as DHA and EPA are different between the baseline of the participants and MedDiet. These n-3 PUFA-derived NAEs and 2-MAGs have been shown to possess anti-inflammatory effects that may be beneficial for metabolic health, as they are known to activate receptors that, due to their pro-lipolytic, insulin-sensitizing and incretin-like actions, are considered metabolically beneficial, including PPARα, GPR119, and TRPV1 [16, 20]. Similarly, the plasmatic concentrations of OEA and 1- and 2-OG, a NAE and 2-MAG respectively, derived from oleic acid, a fatty acid also enriched in the MedDiet interventions, were not modulated by the first MedDiet, but decreased by the CanDiet and reincreased to initial level with the second MedDiet. For most participants, oleic acid intake in their diet prior to the study was similar to CanDiet composition. Consistent with the literature, the palmitic acid-derived NAE (PEA) and MAG (1 and 2-PG) did not significantly respond to the diet. In past studies, PEA has been shown to be unresponsive to dietary modulation [56], and this is possibly due to the fact that mammals can produce significant amounts of de novo palmitic acid to overcome dietary changes [57]. On the other hand, 1–2 PG tended to respond inversely from NAEs and MAGs derived from polyunsaturated fatty acids, especially regarding correlations with the microbiome (Fig. 6). Interestingly, this mediator, which also activates PPARα, was the only eCBome signal to be increased in the plasma following *A. muciniphila* administration to obese individuals [14]. Finally, it is important to emphasize how the two endocannabinoids, AEA and 2-AG, which are derived from arachidonic acid and activate the metabolic syndrome exacerbating receptor CB_1_, but also the anti-inflammatory receptor CB_2_ [15], were not significantly increased by the MedDiet interventions. This observation could be explained by the fact that the MedDiet and CanDiet had no difference in arachidonic acid, 0.13g vs 0.14g. Overall, the endocannabinoids and congeners that were positively modulated by the MedDiet were associated to mechanisms that are assumed to have a beneficial effect on health. Conversely, the decreased levels of MUFA- and n-3 PUFA-derived eCBome mediators that followed the CanDiet, which can be considered as a “Western diet” compared to the MedDiet, might play a role in the metabolically negative effects of such dietary patterns.

Fatty acids produced by the microbiota, i.e., SCFAs and BCFAs, responded differently to the dietary interventions. Acetate, butyrate, and propionate varied little or not at all. They are the most prevalent metabolites produced by the gut microbiota from carbohydrate fermentation [12]. Since the MedDiet contained considerably more fiber compared to the CanDiet, an increase in the production of SCFAs was expected. Moreover, an observational study by Wang and collaborators showed that long-term Mediterranean-style dietary pattern was associated with impact on gut microorganisms associated, among other functions, to SCFA production [4]. However, despite the fact that the microbiota was altered by our short-term dietary interventions, the presence and abundance of the major taxa constituting the intestinal microorganisms of individuals was maintained, thus ensuring metabolic activities. Indeed, two of the most abundant genera in our study cohort are known producers of butyrate (*Faecalibacterium*), and acetate and propionate (*Bacteroides*) [11]. Such high proportion of these taxa may prevent the SCFA modulation by the diet. As for the BCFAs, isovalerate and isobutyrate, they are derived from the fermentation of branched-chain (isoleucine, leucine, and valine) or aromatic (tyrosine and phenylalanine) amino acids, many of which are considered deleterious to intestinal and metabolic health [9]. Accordingly, these amino acids were present in much greater amounts in the CanDiet than in the MedDiet. Indeed, SCFAs and BCFAs were not significantly modulated by the first MedDiet (V2) intervention, suggesting that the food required for their production was comparable between the baseline of the participants and the MedDiet (V2). A strong effect of CanDiet was, however, observed for BCFAs and valerate following the first MedDiet intervention, and this effect was reversed following the second MedDiet (V4) intervention. It is of note that we measured these metabolites in plasma and not in the gut and our measure thus integrates gut metabolite production, their absorption and host metabolic activity. Still, Fig. 6 shows that BCFAs were heavily correlated with the gut microbiota, suggesting a potential role of the microbiome in determining the plasma concentration of these lipid mediators.

Specific gut microbes responded rapidly to short-term MedDiet or CanDiet interventions. *Bacteroides* was the only genera with average relative abundance higher than 5% that was modulated by MedDiet. *Bacteroides* includes potentially beneficial species that could promote mucosal colonization, enforce the epithelial barrier of the gut, and have anti-inflammatory properties [58, 59]. All other modulated taxa had a relative abundance lower than 5%, an observation similar to what was reported after 7 days of antibiotics [28], which further supports the idea that many microbiota modulators affect lower abundance taxa. Bacterial genera that increased with the MedDiet include, among others, *Butyricicoccus* and *Roseburia*, which are negatively associated with obesity clinical markers in the literature [60, 61]. *Buryticicoccus* was previously shown to increase after 4 days of MedDiet [62], and we now demonstrate a similar effect after only 2 days. *Roseburia* has been shown to increase with non-digestible carbohydrates [63], supporting the direct association between these taxa and the diet, and it has previously been negatively associated with type 2 diabetes [64, 65]. *Romboutsia* was positively associated with obesity and increased with the CanDiet in our study [66]. *Subdoligranulum* increased with CanDiet consumption and correlated with BCFAs. The previous literature shows contradictory results regarding this genus, indicating that its involvement in metabolic disorders may be complex. However, it was shown to be associated with positive health impact, like *A. muciniphila* [67], and, accordingly, to be negatively associated with HOMA-IR, although enriched in individual with type 2 diabetes [60, 61]. Interestingly, *Lachnospira* and *Coprococcus 2* were positively correlated with circulating levels of EPA, a PUFA that is a hallmark of the MedDiet. These two taxa were previously found to be associated with the MedDiet score in a large USA cohort [68]. We thus observed strong correlations between taxa and molecules that are coherent with their potential role in metabolic health, be them beneficial or noxious.

Many of the changes induced by the CanDiet were reversed by 48 h of MedDiet, except for *Lactobacillus* and *Ruminococcaceae NK4A214*, which did not recover after the second MedDiet, an observation consistent with their negative association with diets rich in animal protein. *Ruminococcaceae NK4A214* has also been shown to be associated with low HOMA-IR [53, 69]. *Coprococcus 3* was increased by CanDiet, but was not reduced back to its initial relative abundance by the second MedDiet. Similarly, the increase of *Ruminiclostridium* 5 induced by the CanDiet was still observed after the short MedDiet intervention. The decrease in *Coprococcus 3* has previously been associated with a decrease in adiposity [70]. A 1-year-long MedDiet intervention was shown to reduce this genus, indicating that 2-day MedDiet was not long enough to reverse the effect of 14-day CanDiet on this taxon. Interestingly, David and collaborators showed that 2 days of washout is enough to reverse the effect of a 5-day dietary intervention of animal-based diet [3]. While previous studies suggest that gut microbiota come back to baseline composition after a dietary intervention [71], other works in mice show that the change might be irreversible after a long dietary intervention, with a permanent loss of microbiota diversity in mice fed long term with a low microbiota accessible carbohydrate diet [72].

The factors determining the capacity of the gut microbiome to return to a former state after a long-term diet remain to be specifically determined. As specific diets are associated with diseases, it is important to understand after how much time the diet effect on the gut microbiota can be reversed to avoid reaching the point where a deleterious diet causes a permanent disruption of gut microbiota function. In our study, the poor resilience of *Lactobacillus*, *Ruminococcaceae NK4A214*, *Ruminiclostridium 5*, and *Coprococcus 3* to drastic short-term dietary changes could be linked to potential long-lasting effects of Western diets on metabolic health-associated risks [73].

One limitation of the current study is the use of a metataxonomic approach, which does not provide gene and function resolution to the association of gut microbiota with the plasma metabolome. Methodologies providing functional information on the microbiome would provide more precise clues to the mechanisms underlying the interactions between the gut microbiome, the diet, and lipid mediators. It is also important to mention that the correlations observed between bioactive lipids and the gut microbiome in this controlled clinical study do not directly imply causality between correlated variables, although these results do provide insight into the potential relationship between these lipids, the gut microbiome, and diet.

Our findings highlight that a 48-h MedDiet, after either the host usual diet or the short lead-in fully controlled diet period, impacts plasma eCBome mediators in the same way, while the responses of plasma BCFA levels and fecal microbiota composition differ according to the previous diet. This divergent response allows us to hypothesize that the recent lead-in dietary intakes may qualitatively affect the short-term response of the gut microbiota composition, in addition to modifying its metabolic functions in as much as they can be assessed by quantifying BCFAs in the blood. As a consequence, the composition of the host circulating eCBome signals may depend more on the diet than on gut microbiota composition and function, at least in generally healthy individuals.

## Conclusions

Using defined diets that are consumed by all the participants in a cohort with precise anthropometric characteristics, we were able to determine diet-induced modulations of the microbiota and some of its related metabolites, as well as of an increasingly important set of host lipid mediators, and thus deeply investigate the interaction between the microbiome and circulating signals independently of the usual diet of the participants. Altogether, our results suggest that the diet influences both the microbiome and the eCBome in a rapid, direct, and significant way. However, the correlation between microbes and some members of the eCBome do not seem to be directly associated with the diet, confirming that the diet and the gut microbiome may be independent determinants of eCBome signalling [21]. Plasma BCFAs, instead, were clearly associated to both diet and gut microbiota, suggesting that their roles are intimately tied with those of these two related factors. The fact that the effect of a 2-week CanDiet on BCFAs could be reversed by 2 days of MedDiet suggests that some potentially health-detrimental metabolites can be quickly improved with a short-term dietary intervention. On the other hand, the negative effect of a 2-week CanDiet on specific microbiota genera that have been associated with metabolic health were not reversed by 2 days of MedDiet. The diet of the participants in the weeks before the dietary intervention leaves a trace in the response of gut microbiota taxa. These results thus highlight the importance of considering the dietary habits of participants when studying the gut microbiota. Often, studies associating gut microbiota taxa to metabolites or dietary intakes provide results that are contradictory [74]. The complexity of the interactions between microorganisms in the gut ecosystem, and the limitation of computational methods for correlation have been suggested to explain this high variability [75]. Some long-lasting and short-term diet-resilient effects of the previous diet that we observed in our cohort reinforce the hypothesis that the diet is a confounding factor in gut microbiota association studies.

In summary, we suggest several take-home messages from this study. First, a short-term diet plays a crucial role in determining both microbiome composition and circulating lipid mediators. Second, the effect and success of dietary interventions may depend on the initial diversity of the gut microbiome of patients. Third, carefully designed clinical trials with full-feeding periods, designed to address specific hypotheses, can bring studies closer to providing mechanistic insights. Therefore, studies investigating the role of the microbiome or lipid mediators in conditions that can be modulated by the diet should: (1) consider previous short-term dietary intakes in the interpretation of the results, as they could be among the determining factors of their observations, or (2) try to minimize the impact of this factor with a diet stabilization phase.

However, although several of the taxa found here to be modulated in response to dietary changes have been previously associated to metabolic diseases, the uncertainty that their role may not be mostly related to the diet remains, possibly also due to us having recruited healthy and young individuals for this study. In this work, the use of a cohort of healthy young individuals provided crucial information on our understanding of the relationship between diet, the gut microbiome, and lipid mediators in a non-pathological context. Future studies should address similar questions in cohorts suffering from different metabolic conditions to determine if the response to short-term dietary interventions is comparable between healthy and non-healthy individuals. This experimental approach would contribute to a transition from observational approaches to more mechanistic clinical studies and possibly open new avenues for the development of novel therapies for metabolic disorders.

## Supplementary Information

**Additional file 1:** **Figure S1. Metataxonomic sequencing library size.** Total sequence counts of the microbiota samples regrouped by study visits.

**Additional file 2:** **Figure S2. Microbiota diversity between visits.** Microbiota alpha diversity measured on all sequence variants by A) Simpson’s index (1-D) and B) Shannon index between visits of the intervention study. Euclidean distance of microbiota profile including all sequence variants between baseline and each visit of the study for each participant in relation with their alpha diversity represented by C) Shannon index or D) total observed ASVs at baseline. Regression lines were drawn for each visit. Slope of line (lm) were significant for microbiota distance measurements with p-values < 0.05. Spearman coefficients are displayed on the legend. Red represents the distance between the microbiota profile of baseline (V1) and the first MedDiet (V2), black of baseline (V1) and CanDiet (V3) and gray of baseline (V1) and the second MedDiet (V4). Significance was set at p<0.05 (*), p<0.01 (**) and p<0.001 (***).

**Additional file 3:** **Figure S3. Multiple factor analysis (MFA) on microbiota profile.** A) Loading plot representing the contribution of taxonomic ranks of the gut microbiota to the variability between individuals at different visits. Taxa representing less than 1% in every sample have been filtered out. The ellipses represent the 95% confidence interval of the mean of points as computed with the FactoMineR package for the effect of B) diet, C) participants and D) the five clusters of samples distinguished from hierarchical clustering of the PCA. A sixth cluster containing only two samples was excluded from cluster analysis. Barplot representing the contribution of the top 15 variables to the MFA for E) dimension 1 and F) dimension 2.

**Additional file 4:** **Figure S4. Distribution of participants visits in clusters from microbiota profiles.** Heatmap representing the number of visits per participant in each cluster of the microbiota MFA.

**Additional file 5:** **Figure S5. Clustering of bioactive lipid profiles.** Principal component analysis (PCA) on bioactive lipid profiles. A) Loading plot representing the contribution of lipids from each category to the variability between individuals at different visits. Ellipses show the effect of B) diet and C) individuals on samples. The ellipses represent the 95% confidence interval of the mean of points as computed with the FactoMineR package for the effect of B) diet, C) participants and D) the five clusters of samples distinguished from hierarchical clustering of the PCA. E) Hierarchical clustering on principal components (HCPC) of lipid profile of individuals at different visits. Variables contributing to the difference between the clusters are displayed on the dendrogram. A colored bar is printed below the dendrogram represents the visit to which the sample belongs. F) Heatmap of the lipid concentration for each sample divided by cluster and molecule category. For better visualization, the concentration values were centered around the mean for each metabolite.

**Additional file 6:** **Figure S6.** Heatmap illustrating the FDR-corrected Spearman correlations between HEI score of participants before the intervention study and gut microbiota genera relative abundances, Simpson’s diversity index, Shannon diversity index and plasmatic lipid concentrations at baseline (V1). Genera representing less than 1% in every sample have been filtered out. No feature was significant after FDR correction.

**Additional file 7:** **Supplementary Table S1.** Results of statistical analysis for metataxonomic data at all taxonomical ranks. Results for the ANOVA and post-hoc tests before and after FDR correction are shown for phylum, class, order, family, genera, species and amplicon sequence variants (ASV). Mean values of each variables for all four visits are also shown.

## Data Availability

Raw sequencing reads are available in SRA (PRJNA810015).
