# Supplementary material for: The diet rapidly and differentially affects the gut microbiota and host lipid mediators in a healthy population
Source: Microbiome. 2023 Feb 11;11:26. doi: 10.1186/s40168-023-01469-2 (PMC9921707; doi:10.1186/s40168-023-01469-2)
Supplement: Supplementary file 2 — Additional file 1: Figure S1. Metataxonomic sequencing library size. Total sequence counts of the microbiota samples regrouped by study visits. [file 40168_2023_1469_MOESM1_ESM.pdf]

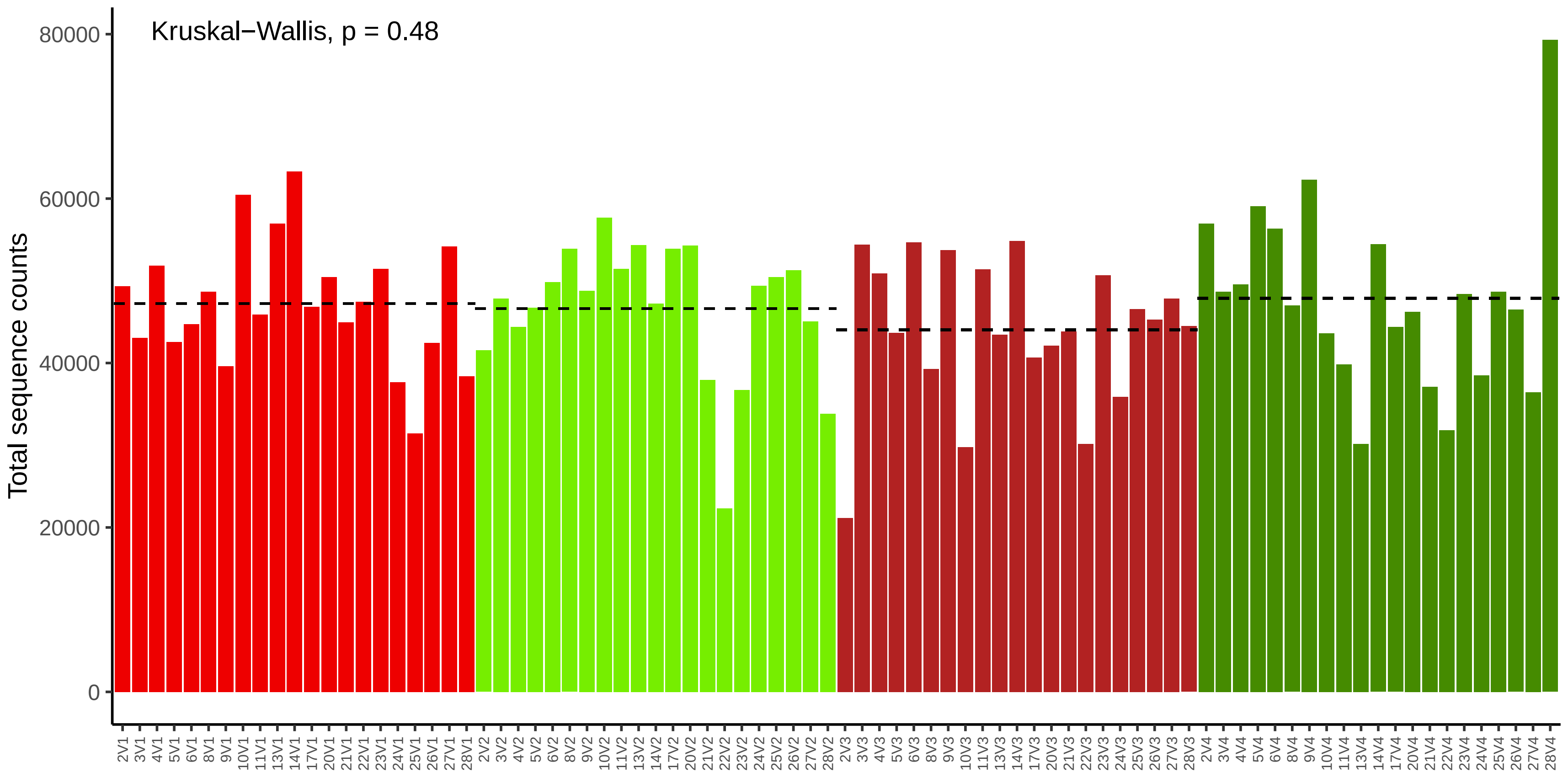
Kruskal−Wallis, p = 0.48
Total sequence counts
0
20000
40000
60000
80000
2V1 3V1 4V1 5V1 6V1 8V1 9V1 10V1 11V1 13V1 14V1 17V1 20V1 21V1 22V1 23V1 24V1 25V1 26V1 27V1 28V1
2V2 3V2 4V2 5V2 6V2 8V2 9V2 10V2 11V2 13V2 14V2 17V2 20V2 21V2 22V2 23V2 24V2 25V2 26V2 27V2 28V2
2V3 3V3 4V3 5V3 6V3 8V3 9V3 10V3 11V3 13V3 14V3 17V3 20V3 21V3 22V3 23V3 24V3 25V3 26V3 27V3 28V3
2V4 3V4 4V4 5V4 6V4 8V4 9V4 10V4 11V4 13V4 14V4 17V4 20V4 21V4 22V4 23V4 24V4 25V4 26V4 27V4 28V4
