# Supplementary figures and images for: The diet rapidly and differentially affects the gut microbiota and host lipid mediators in a healthy population
Source: Microbiome. 2023 Feb 11;11:26. doi: 10.1186/s40168-023-01469-2 (PMC9921707; doi:10.1186/s40168-023-01469-2)

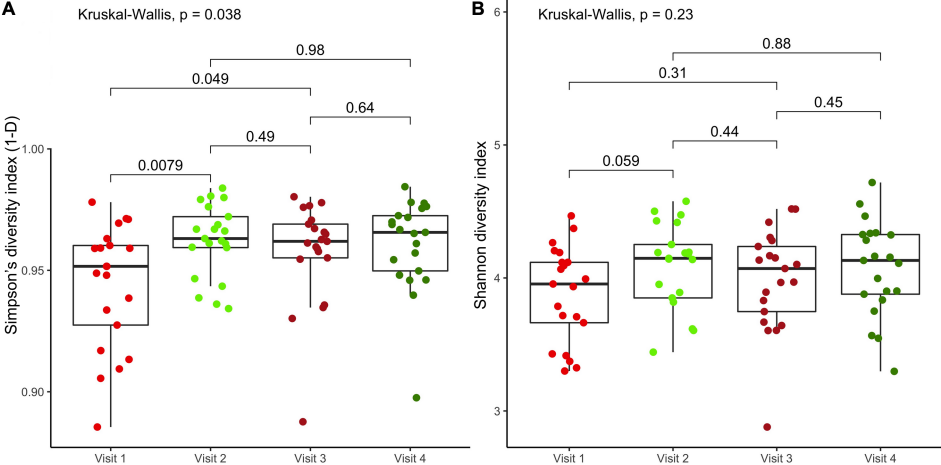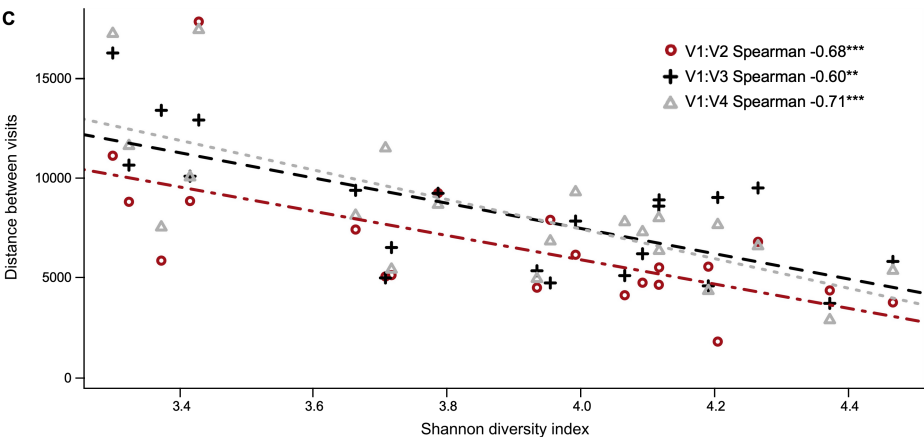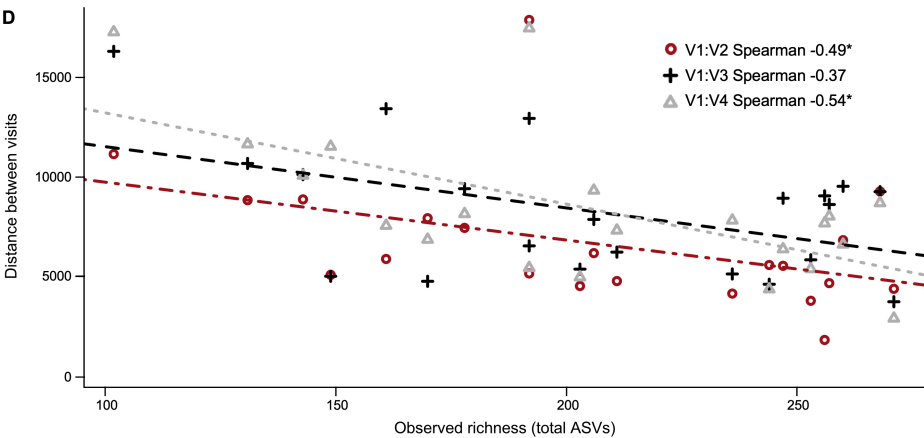

Supplement: Supplementary file 3 — Additional file 2: Figure S2. Microbiota diversity between visits. Microbiota alpha diversity measured on all sequence variants by A) Simpson’s index (1-D) and B) Shannon index between visits of the intervention study. Euclidean distance of microbiota profile including all sequence variants between baseline and each visit of the study for each participant in relation with their alpha diversity represented by C) Shannon index or D) total observed ASVs at baseline. Regression lines were drawn for each visit. Slope of line (lm) were significant for microbiota distance measurements with p-values < 0.05. Spearman coefficients are displayed on the legend. Red represents the distance between the microbiota profile of baseline (V1) and the first MedDiet (V2), black of baseline (V1) and CanDiet (V3) and gray of baseline (V1) and the second MedDiet (V4). Significance was set at p<0.05 (*), p<0.01 (**) and p<0.001 (***). [file 40168_2023_1469_MOESM2_ESM.pdf]

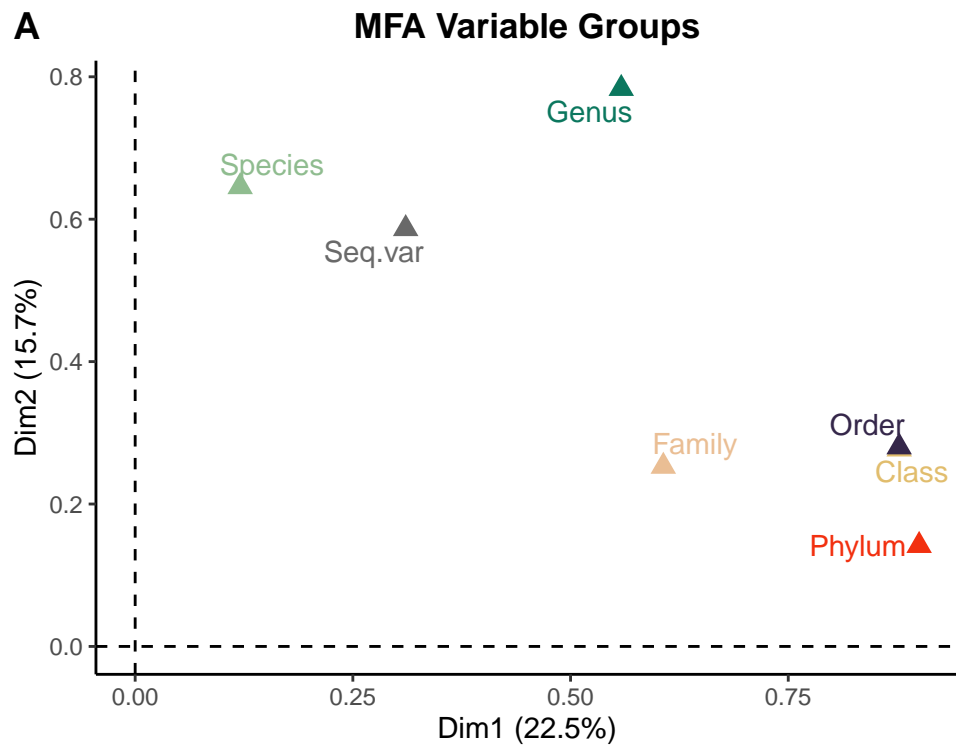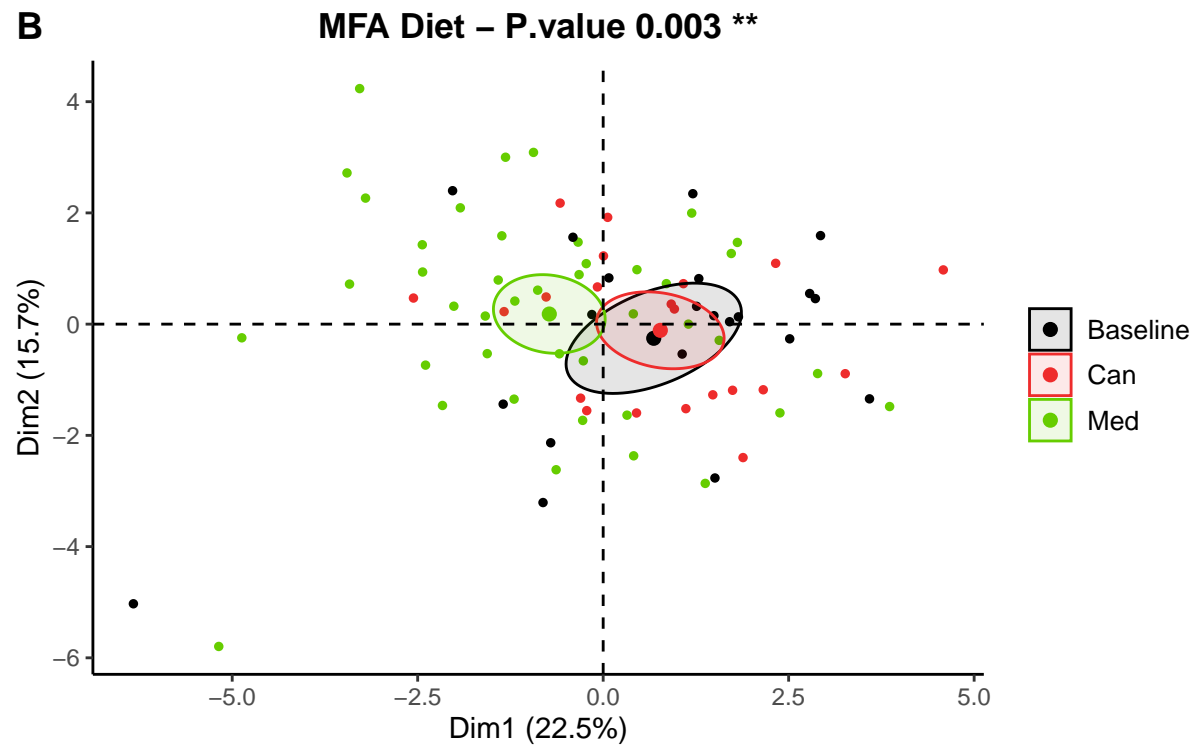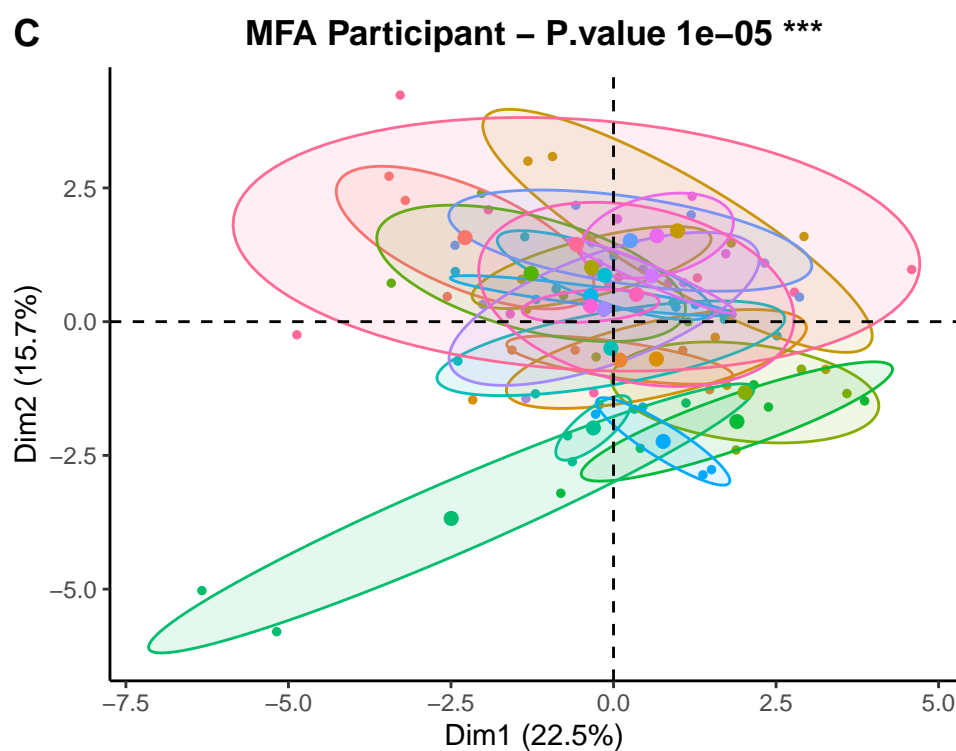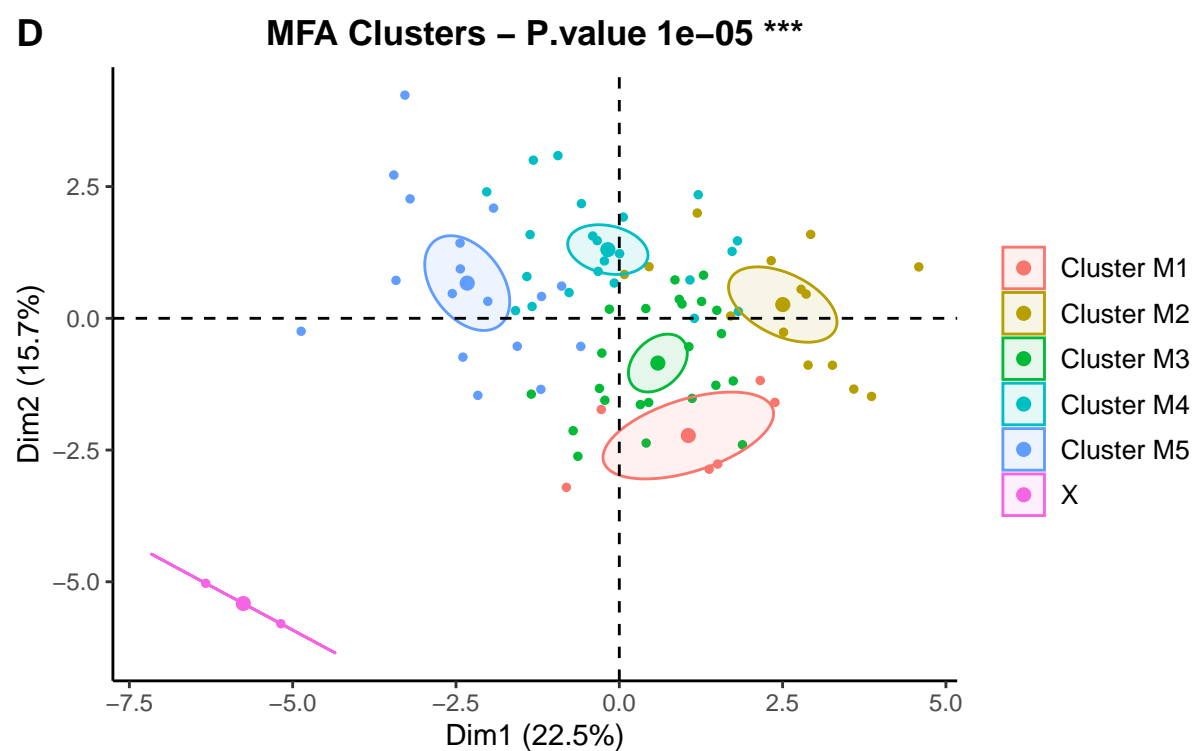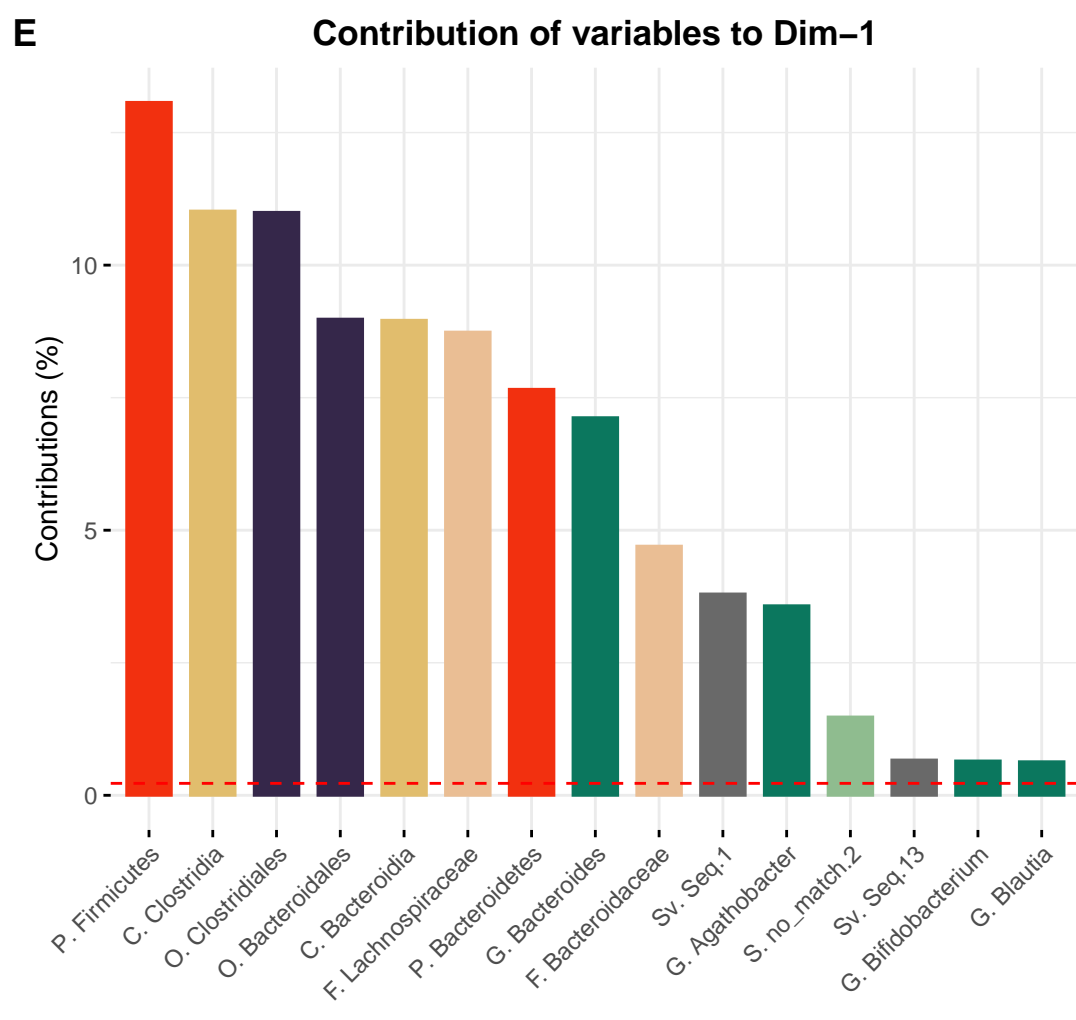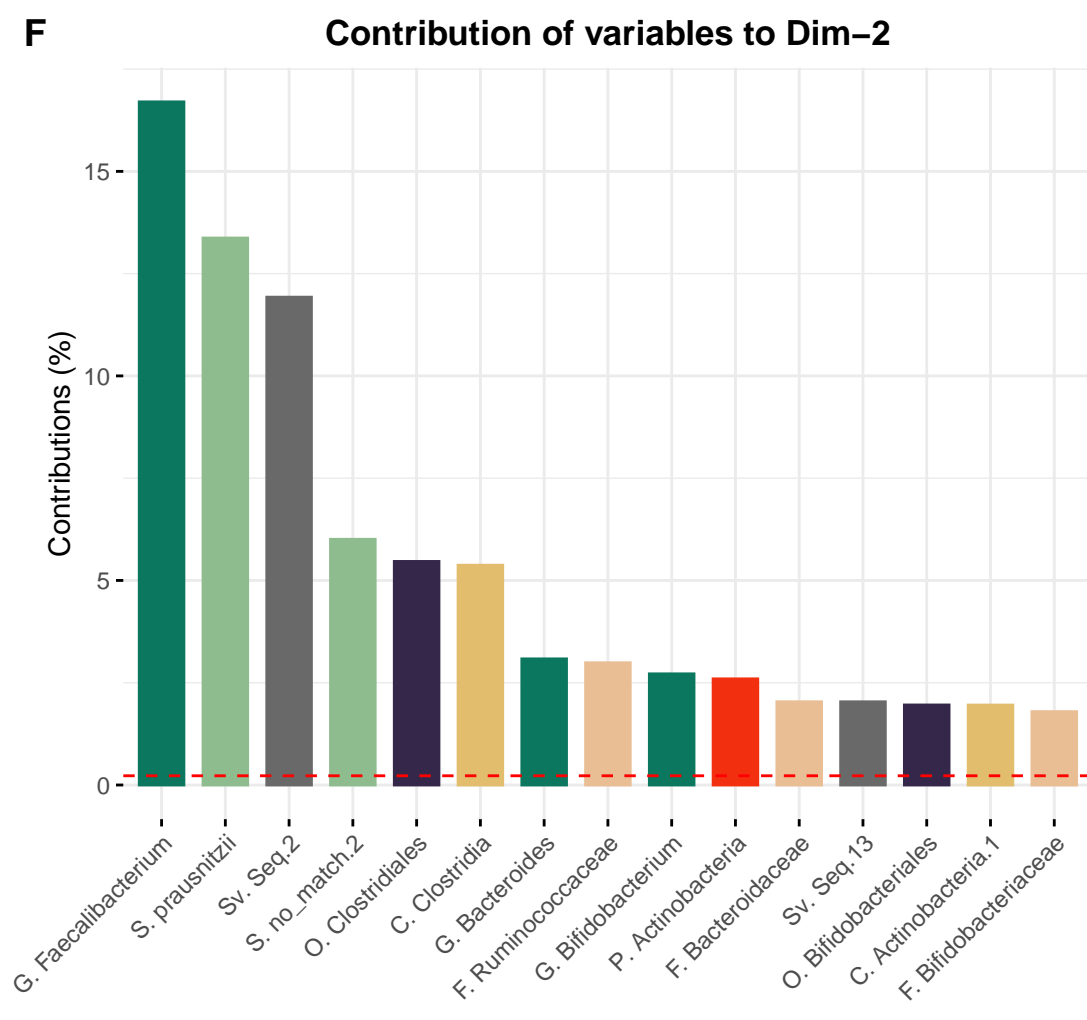

Supplement: Supplementary file 4 — Additional file 3: Figure S3. Multiple factor analysis (MFA) on microbiota profile. A) Loading plot representing the contribution of taxonomic ranks of the gut microbiota to the variability between individuals at different visits. Taxa representing less than 1% in every sample have been filtered out. The ellipses represent the 95% confidence interval of the mean of points as computed with the FactoMineR package for the effect of B) diet, C) participants and D) the five clusters of samples distinguished from hierarchical clustering of the PCA. A sixth cluster containing only two samples was excluded from cluster analysis. Barplot representing the contribution of the top 15 variables to the MFA for E) dimension 1 and F) dimension 2. [file 40168_2023_1469_MOESM3_ESM.pdf]

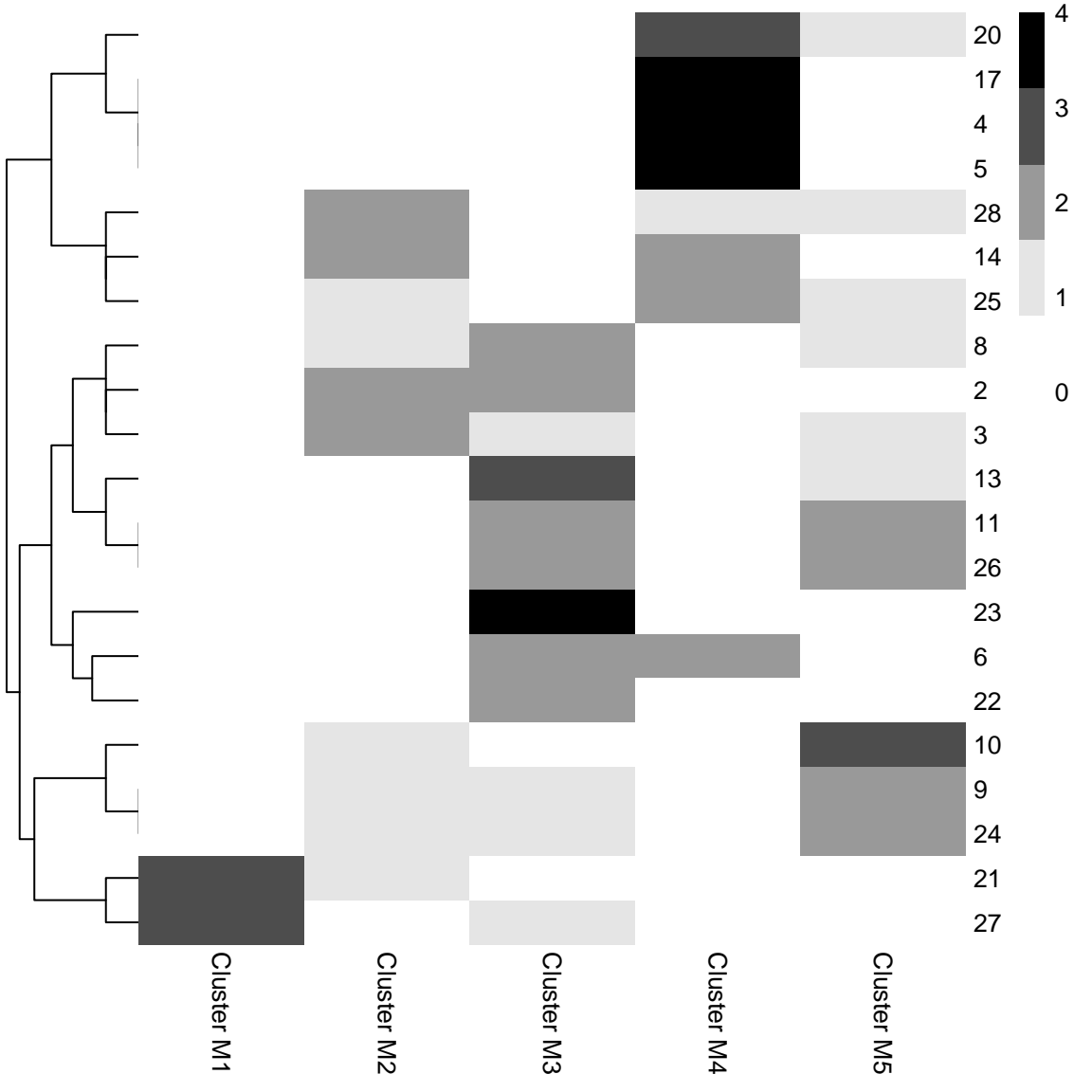

Supplement: Supplementary file 5 — Additional file 4: Figure S4. Distribution of participants visits in clusters from microbiota profiles. Heatmap representing the number of visits per participant in each cluster of the microbiota MFA. [file 40168_2023_1469_MOESM4_ESM.pdf]

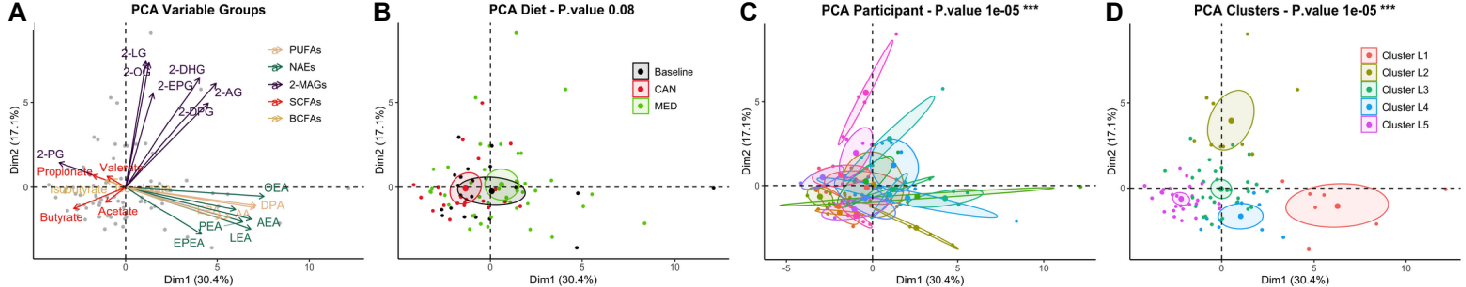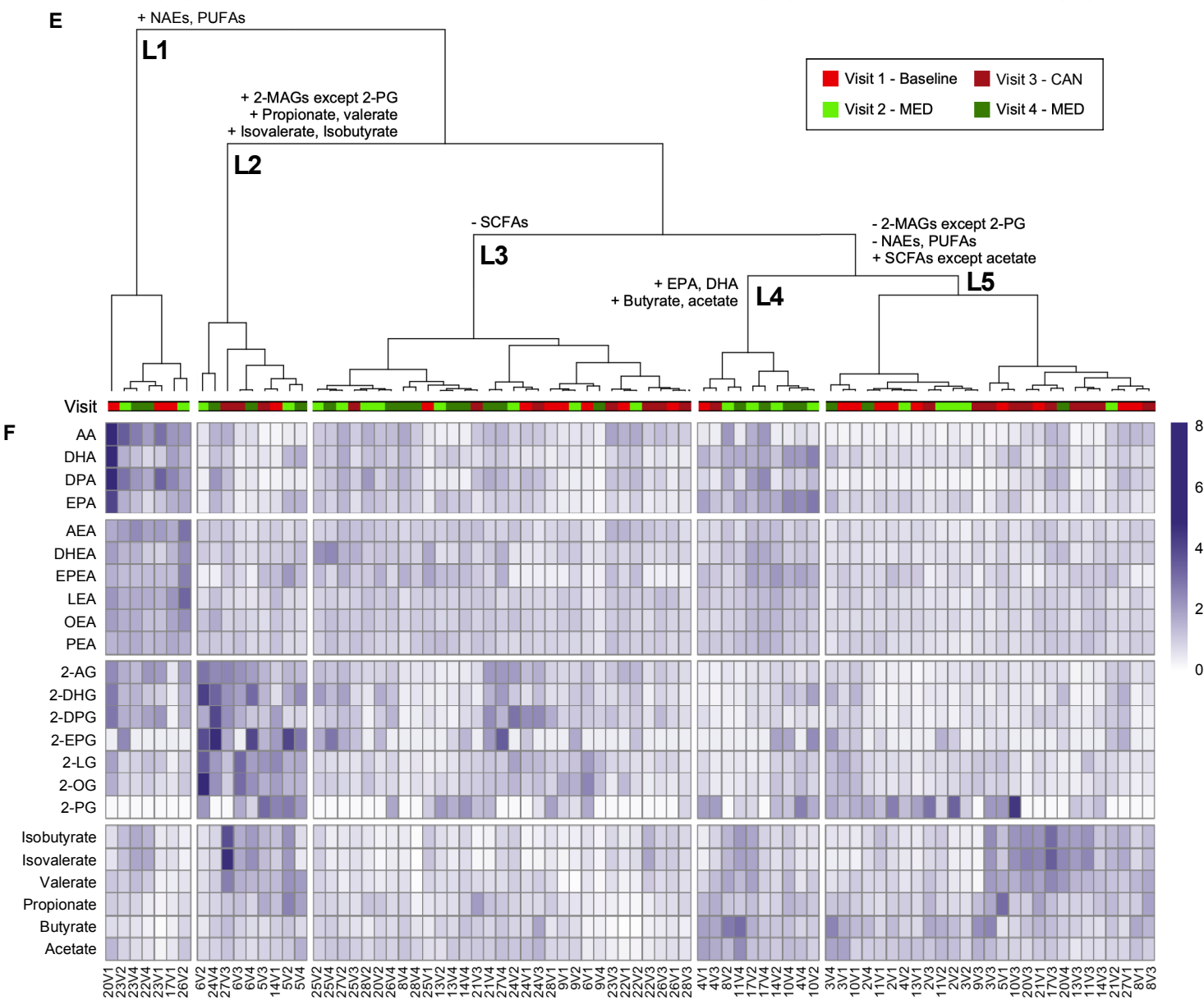

Supplement: Supplementary file 6 — Additional file 5: Figure S5. Clustering of bioactive lipid profiles. Principal component analysis (PCA) on bioactive lipid profiles. A) Loading plot representing the contribution of lipids from each category to the variability between individuals at different visits. Ellipses show the effect of B) diet and C) individuals on samples. The ellipses represent the 95% confidence interval of the mean of points as computed with the FactoMineR package for the effect of B) diet, C) participants and D) the five clusters of samples distinguished from hierarchical clustering of the PCA. E) Hierarchical clustering on principal components (HCPC) of lipid profile of individuals at different visits. Variables contributing to the difference between the clusters are displayed on the dendrogram. A colored bar is printed below the dendrogram represents the visit to which the sample belongs. F) Heatmap of the lipid concentration for each sample divided by cluster and molecule category. For better visualization, the concentration values were centered around the mean for each metabolite. [file 40168_2023_1469_MOESM5_ESM.pdf]

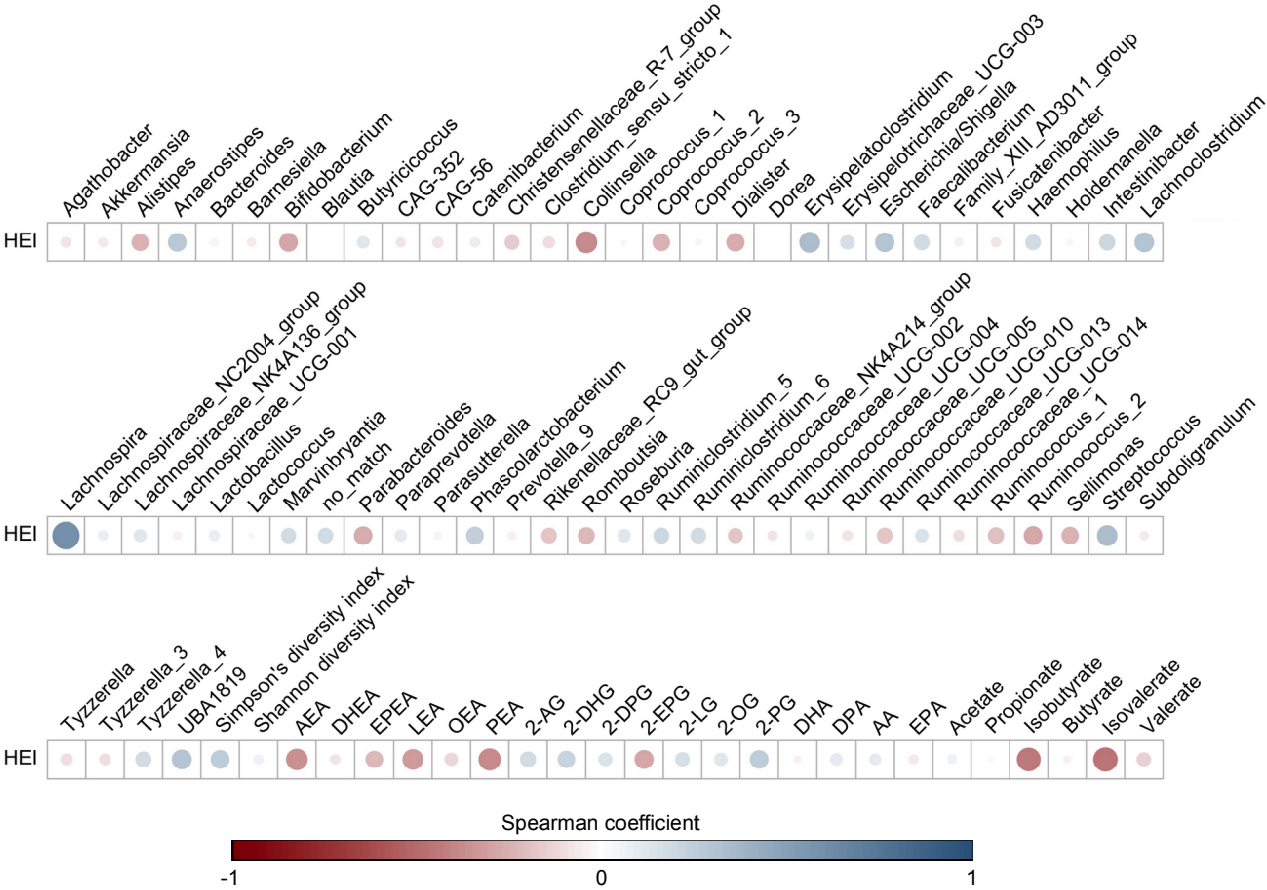

Supplement: Supplementary file 7 — Additional file 6: Figure S6. Heatmap illustrating the FDR-corrected Spearman correlations between HEI score of participants before the intervention study and gut microbiota genera relative abundances, Simpson’s diversity index, Shannon diversity index and plasmatic lipid concentrations at baseline (V1). Genera representing less than 1% in every sample have been filtered out. No feature was significant after FDR correction. [file 40168_2023_1469_MOESM6_ESM.pdf]
